# The motor system is exceptionally vulnerable to absence of the ubiquitously expressed superoxide dismutase-1

**DOI:** 10.1093/braincomms/fcad017

**Published:** 2023-01-27

**Authors:** Julien H Park, Ulrika Nordström, Konstantinos Tsiakas, Isil Keskin, Christiane Elpers, Manoj Mannil, Raoul Heller, Melinda Nolan, Salam Alburaiky, Per Zetterström, Maja Hempel, Ulrike Schara-Schmidt, Saskia Biskup, Petra Steinacker, Markus Otto, Jochen Weishaupt, Andreas Hahn, René Santer, Thorsten Marquardt, Stefan L Marklund, Peter M Andersen

**Affiliations:** Department of Clinical Sciences, Neurosciences, Umeå University, 901 87 Umeå, Sweden; Department of General Paediatrics, University of Münster, 48149 Münster, Germany; Department of Clinical Sciences, Neurosciences, Umeå University, 901 87 Umeå, Sweden; Department of Paediatrics, University Medical Centre Hamburg-Eppendorf, 20251 Hamburg, Germany; Department of Medical Biosciences, Pathology, Umeå University, 901 85 Umeå, Sweden; Department of General Paediatrics, University of Münster, 48149 Münster, Germany; Clinic for Radiology, University Hospital Münster, WWU University of Münster, 48149 Münster, Germany; Starship Children’s Health, Auckland City Hospital, Auckland 1142, New Zealand; Starship Children’s Health, Auckland City Hospital, Auckland 1142, New Zealand; Starship Children’s Health, Auckland City Hospital, Auckland 1142, New Zealand; Department of Medical Biosciences, Clinical Chemistry, Umeå University, 901 87 Umeå, Sweden; Department of Paediatrics, University Medical Centre Hamburg-Eppendorf, 20251 Hamburg, Germany; Current address: Institute of Human Genetics, University Hospital Heidelberg, 69120 Heidelberg, Germany; Department of Paediatric Neurology, University Hospital Essen, 39081 Essen, Germany; CeGAT GmbH and Praxis für Humangenetik Tübingen, 72076 Tübingen, Germany; Department of Neurology, Martin-Luther-University Halle-Wittenberg, 06120 Halle (Saale), Germany; Department of Neurology, Martin-Luther-University Halle-Wittenberg, 06120 Halle (Saale), Germany; Division for Neurodegenerative Diseases, Department of Neurology, Medical Faculty Mannheim, University of Heidelberg, 68167 Mannheim, Germany; Department of Child Neurology, Justus Liebig University, 35392 Giessen, Germany; Department of Paediatrics, University Medical Centre Hamburg-Eppendorf, 20251 Hamburg, Germany; Department of General Paediatrics, University of Münster, 48149 Münster, Germany; Department of Medical Biosciences, Clinical Chemistry, Umeå University, 901 87 Umeå, Sweden; Department of Clinical Sciences, Neurosciences, Umeå University, 901 87 Umeå, Sweden

**Keywords:** oxygen toxicity, infantile motor neuron disease, ALS, spasticity, SOD1

## Abstract

Superoxide dismutase-1 is a ubiquitously expressed antioxidant enzyme. Mutations in *SOD1* can cause amyotrophic lateral sclerosis, probably via a toxic gain-of-function involving protein aggregation and prion-like mechanisms. Recently, homozygosity for loss-of-function mutations in *SOD1* has been reported in patients presenting with infantile-onset motor neuron disease. We explored the bodily effects of superoxide dismutase-1 enzymatic deficiency in eight children homozygous for the p.C112Wfs*11 truncating mutation. In addition to physical and imaging examinations, we collected blood, urine and skin fibroblast samples. We used a comprehensive panel of clinically established analyses to assess organ function and analysed oxidative stress markers, antioxidant compounds, and the characteristics of the mutant Superoxide dismutase-1. From around 8 months of age, all patients exhibited progressive signs of both upper and lower motor neuron dysfunction, cerebellar, brain stem, and frontal lobe atrophy and elevated plasma neurofilament concentration indicating ongoing axonal damage. The disease progression seemed to slow down over the following years. The p.C112Wfs*11 gene product is unstable, rapidly degraded and no aggregates were found in fibroblast. Most laboratory tests indicated normal organ integrity and only a few modest deviations were found. The patients displayed anaemia with shortened survival of erythrocytes containing decreased levels of reduced glutathione. A variety of other antioxidants and oxidant damage markers were within normal range. In conclusion, non-neuronal organs in humans show a remarkable tolerance to absence of Superoxide dismutase-1 enzymatic activity. The study highlights the enigmatic specific vulnerability of the motor system to both gain-of-function mutations in *SOD1* and loss of the enzyme as in the here depicted infantile superoxide dismutase-1 deficiency syndrome.

## Teaser

Park *et al*. report on eight patients homozygous for the *SOD1* mutation p.C112Wfs*11. By characterizing the clinical and biochemical phenotypes associated with a loss of Superoxide dismutase-1 enzymatic function, they highlight the specific vulnerability of the motor system to both gain-of-function mutations in *SOD1* and complete loss of the enzyme.

## Graphical Abstract

**Graphical abstract:**
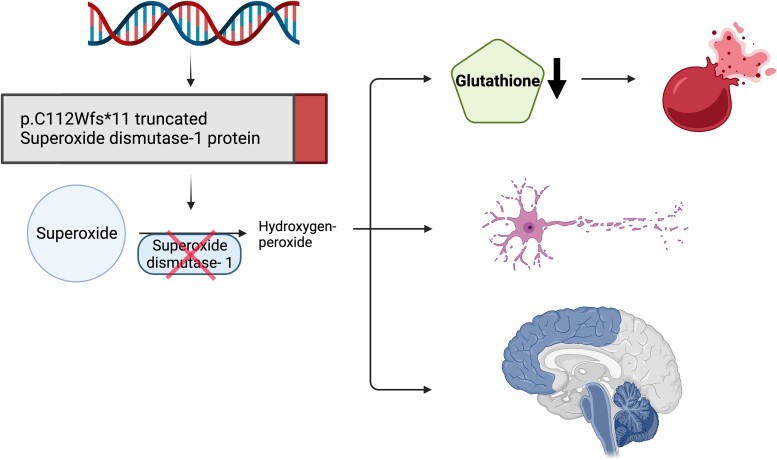

## Introduction

Under aerobic conditions, derivatives of molecular oxygen known as reactive oxygen species (ROS), are generated in vivo. ROS can have detrimental effects on biomolecules and virtually all organ systems but also serve as signalling molecules.^1,2^ An elaborate defence system, comprising both enzymes and low-molecular-weight compounds, protects the human body against ROS.^3^ Among the enzymes, superoxide dismutases represent the main defence against damage caused by superoxide anion radicals (O_2_^•−^).^4^ The three known human isoforms are characterized by distinct biochemical properties and localizations: SOD2 (MnSOD) is located in the mitochondrial matrix;^5^ SOD3 (extracellular SOD, EC-SOD) is secreted into the extracellular space;^6^ and SOD1 (Cu, Zn-SOD) is mainly located in the cytosol but is also found in the mitochondrial intermembrane space and in the nucleus.^7,8^ SOD1 is constitutively and ubiquitously expressed, has been found in all investigated human tissues and cell types^9^ and is considered to be pivotal in intracellular superoxide metabolism.^4^

The concentrations of SOD1 are highest in the liver and kidney, whereas the levels in the CNS are moderate. Nevertheless, human diseases related to impaired SOD1 function almost exclusively show nervous system-related manifestations. Mutations in *SOD1* were first identified as a cause of amyotrophic lateral sclerosis (ALS)^10,11^ in 1993. Most are inherited as a Mendelian dominant trait, suggesting a gain-of-function to be the disease mechanism. The gain-of-toxic-function mechanism was further supported by the finding that some *SOD1* mutations found in ALS patients had normal enzymatic activity,^12^ that transgenic mice overexpressing human *SOD1* mutations found in ALS patients develop ALS-like paralysis with loss of motor neurons, that mutant SOD1 aggregates in affected cells in ALS patients,^13^ that the increased aggregation propensity of mutant SOD1 correlate with disease severity,^14^ and the identification of prion-like propagation and transmission of misfolded SOD1 species.^15–17^ Together, these findings support a prion hypothesis for SOD1-mediated ALS in parallel with similar findings in Alzheimer’s and Parkinson’s disease.

Early on, the role of reduced SOD1 activity in the pathogenesis of ALS was debated.^18^ However, the findings of normal enzymatic activity in patients with the p.D91A and six other mutations,^12^ the lack of correlation between SOD1 enzymatic activity and disease progression or age of onset,^12^ and the fact that SOD1 knockout mice do not develop ALS-like disease, contributed to the general view that reduced SOD1 activity does not cause ALS. Based on these considerations, silencing of *SOD1*, i.e. the suppression of SOD1 protein expression (and consequently enzyme activity), has been proposed as a precision medicine therapeutic approach to ALS.^19,20^

Recently, we identified a novel truncating homozygous variant c.335dupG, p.C112Wfs*11 (hereafter referred to as p.C112X) devoid of SOD1 enzyme activity and a severe infantile-onset neurological phenotype in two children from consanguineous Afghan families.^21,22^ To further delineate the phenotype, we here report a cohort of eight patients homozygous for C112X. We performed clinical, biochemical, and imaging phenotyping, allowing a characterization of the resulting syndrome. The findings have implications for our understanding of superoxide homeostasis in general but also for understanding the pathogenesis of ALS and the ongoing gene therapy trials aimed at suppressing SOD1.

## Materials and methods

### Genetic studies

A detailed methodology for genetic studies in Patients 1 and 2 has been reported previously.^21,22^ All participants included in this study received exome sequencing, targeted Sanger sequencing of *SOD1*, or both, as outlined previously.^21,22^

### Clinical assessment and imaging studies

Clinical data were gathered from previous clinical reports and by follow-up examination for each patient. Patients 1, 2, 3, 4, and 5, as well as 7 and 8 were assessed by the same study investigators in addition to frequent, non-study-related assessments that were part of their standard clinical management. Assessment of Patient 6 was based on clinical reports and video conference interviews due to current restrictions amid the ongoing SARS-CoV-2 pandemic. MRI data were available for seven of the patients reported here. MRI data sets were anonymized using Horos version 3.3.6 (Nimble Co LLC d/b/a Purview, Annapolis, MD USA) and were evaluated with blinding toward the clinical data. Data from unrelated individuals with neurodevelopmental disorders were included as control data sets. All imaging data sets were evaluated by the same neuroimaging specialist (M.M.).

### Sample collection and processing

Venous blood was collected using the Sarstedt S-Monovette® collection system (Sarstedt AG & Co., Nümbrecht, Germany) in either Na-heparin, Li-heparin, K-EDTA or serum tubes depending on the downstream analyses. After blood drawing, samples were placed on ice and processed immediately. For analysis of organ function parameters, samples were centrifuged for 10 min at 2000 × g and 4°C followed by separation of plasma and sediment and stored at −80°C until they were processed further.

### Clinical chemistry

The analyses of the clinically established plasma markers of organ function were carried out at the clinical chemistry laboratory of the University Hospital in Umeå, Sweden. B-HbA1c, as well as analysis for Hb variants, was analysed by HPLC (Tosoh G11, Tokyo, Japan). No haemoglobin variants that might influence the results were detected. P-Fructosamine, a measure of plasma protein glycation, was analysed with the Diazyme Glycated Serum Protein Assay at the Department of Medical Sciences, Clinical Chemistry, Uppsala University (courtesy Prof. Anders Larsson).

### Neurofilament light chain and phosphorylated neurofilament heavy chain

Neurofilament levels were measured in EDTA-treated plasma samples as previously described.^23,24^

### Immunoblots and misELISA

All materials, apparatus, and software for immunoblots were from Bio-Rad Laboratories (Hercules, CA, USA) unless stated elsewhere. Immunoblots of erythrocyte lysates were performed as previously described.^25^ Immunoblot analysis of soluble and detergent-insoluble fractions of SOD1 in patient skin biopsy-derived fibroblast cultures was carried out essentially as described before.^26^ We used inhouse produced polyclonal rabbit anti-peptide antibodies specifically targeting aa 5–21 (5 µg/ml), aa 25–40 (1 µg/ml) of the SOD1 protein sequence, and the neo-peptide sequences of the p.G127X (2 µg/ml) and the p.C112X (10 µg/ml) mutant proteins. Detailed experimental procedures are available in the Supplementary material.

### Redox metabolites and parameters of oxidative damage

#### LC–MS analysis of GSH, GSSG, and ascorbate

Samples for glutathione metabolite analysis were prepared by mixing Na-heparin-anticoagulated whole blood with freshly prepared 10% meta-phosphoric acid (MPA) solution containing 2 mmol/l diethylenetriaminepentaacetic acid (DTPA) at a ratio of 1:5 and incubated for 5 min on ice. Following 10 min of centrifugation at 2000 × g and 4° C, the supernatant was stored at −80° C. Before analysis, the acidified whole blood samples were thawed, and 50 µL of each sample was mixed with 150 µL of extraction solution (1.67% MPA, internal standard (IS-)GSH at 3.33 µM). For ascorbate analysis, 1 ml of Na-heparin plasma was mixed with 1 ml of a freshly prepared 10% MPA solution containing 2 mmol/l DTPA and vortexed, followed by an incubation of 5 min on ice. After 10 min of centrifugation at 2000 × g and 4°C, the supernatant was frozen at −80°C until further analysis. The acidified plasma samples were thawed, and 50 µL was mixed with 150 µL of extraction solution (3.1% MPA, IS-ASA 3.33 µM). The analyses were performed as previously described,^27,28^ and modifications for ASA, IS-ASA and DHA were added to the method.

#### ELISA analysis of 8-isoprostane and 8-OHdG

For analysis of 8-isoprostane, a marker of lipid peroxidation, and 8-hydroxy-2-deoxyguanosine (8-OHdG), the result of oxidative damage to DNA and RNA, urine samples were collected and centrifuged at 2000 × g before being stored at −80°C until further analysis. Samples were analysed with commercially available competitive immunoenzymatic assays (Abcam, Cambridge, United Kingdom, ab2175819/ab 201734) according to the manufacturer’s protocols. Control samples for adult individuals were obtained from healthy volunteers. Paediatric control samples were collected from female and male children aged 5–11 years undergoing evaluation for nocturnal enuresis in the absence of infections or other underlying diseases. Controls for 8-isoprostane were diluted twofold prior to analysis, while samples for 8-OHdG analysis were diluted 20-fold. All measurements were performed in triplicate. The mean of the values for each patient was computed for downstream analysis.

#### Statistical analyses

Statistical analyses were performed using GraphPad Prism (Version 9.2.0) for Mac (GraphPad Software, San Diego, CA, USA). The means of continuous variables were compared using the Mann–Whitney U or Kruskal–Wallis tests, followed by Dunn’s *post hoc* test. *P* values < 0.05 were considered to be statistically significant. The results are presented as the mean ± standard deviation. *P* < 0.05 *, *P* < 0.01 **, *P* < 0.001 ***.

### Data availability

Reasonable data sharing requests are made in writing through the corresponding author (email) and require a formal data sharing agreement adhering to the European Union General Data Protection Regulation (GDPR) 2016/679. Data sharing agreements must include details on how the data will be stored, who will have access to the data and intended use of the data, and agreements as to the allocation of intellectual property.

## Results

### Homozygosity for *SOD1* c.335dupG, p.C112Wfs*11 is associated with a severe neurological phenotype and elevated plasma neurofilament levels

Following the identification of the first two cases exhibiting a severe neurodevelopmental phenotype associated with p.C112X, a call for additional patients was issued through an email-based network of paediatric neurologists in Germany (Erhebung Seltener Neurologischer Erkrankungen im Kindesalter, ESNEK)^29^ and Sweden and ALS neurologists across Europe (www.ENCALS.eu) and North America. A collaborative network of researchers was formed with the aim of identifying additional patients. A total of eight homozygous patients (C112X^Hom^) was identified in five families, including the two first reported^21,22^ (3 males, ages 3, 8, and 10 years; 5 females, ages 4, 4, 5, 9, and 13 years). In addition, 13 heterozygous relatives (parents and siblings, C112X^Het^) were included [eight parents (four males and four females) and four siblings (one male and three females), demographic data in Supplementary Table 1]. An overview of the phenotype observed in the C112X^Hom^ patients is given in Table 1 with additional information in the Supplementary material (Supplementary phenotype data and Supplementary Table 2). Of note, no asymptomatic C112X SOD1^Hom^ individual was found in any of the families, and none of the heterozygous carriers showed an overt pathological phenotype. The parents of the children reported no family members with adult-onset ALS or a condition with similar symptoms.

**Table 1:** Clinical presentation of infantile SOD1 deficiency syndrome (ISODDES)^a^

	Patient 1	Patient 2	Patient 3	Patient 4	Patient 5	Patient 6	Patient 7	Patient 8
Family	A	B	C	D	D	A	E	E
Sex	Male	Female	Female	Female	Male	Female	Female	Male
Age	8 years	4 years	4 years	13 years	10 years	5 years	9 years	3 years
Age of onset	9 months	6 months	10 months	9–12 months	∼ 10 months	6–7 months	5 months	5 months
Upper motor neuron symptoms	Generalized spasticity	Generalized spasticity	Generalized spasticity	Generalized spasticity	Generalized spasticity	Generalized spasticity	Generalized spasticity	Generalized spasticity
	Hyperreflexia	Hyperreflexia	Hyperreflexia	Hyperreflexia	Hyperreflexia			
	Loss of abdominal reflex			Loss of abdominal reflex	Loss of abdominal reflex			
Lower motor neuron symptoms	Axial hypotonia	Axial hypotonia	Axial hypotonia	Axial hypotonia	Axial hypotonia	Axial hypotonia	Axial hypotonia	Axial hypotonia
	Mild muscular atrophy		Generalized muscular atrophy	Generalized muscular atrophy	Generalized muscular atrophy			
			Intermittent fasciculations	Intermittent fasciculations	Intermittent fasciculations			
Bulbar symptoms	Spastic dysarthria	Spastic dysarthria	Spastic dysarthria	Spastic dysarthria	Spastic dysarthria	N/A	Multiphase dysphagia	Multiphase dysphagia
	Dysphagia	Dysphagia	Dysphagia	Mild dysphagia	Dysphagia			
		Sialorrhea	Sialorrhea	Tongue atrophy & fasciculations	Sialorrhea			
			Tongue atrophy & fasciculations		Tongue atrophy & fasciculations			
Frontal lobe symptoms	Glabellar tap sign	Glabellar tap sign	–	Inadequate fits of laughter, onset at 13 years of age	Glabellar tap sign	N/A	–	–
		Inadequate crying			Inadequate fits of laughter, onset at 9 years			
Excessive startle	+	–	–	–	–	–	Distress induced self-resolving tonic decerebrate posture	Distress induced self-resolving tonic decerebrate posture
Ocular symptoms	–	–	Bilateral optic atrophy	–	–	N/A	Bilateral optic atrophy	–
Dysmorphic symptoms	Low set, posteriorly rotated ears	Broad nasal bridge	–	Low set ears	Low set ears	Low set ears	–	Simplified outer helix of the right ear
								Hydrocephalus
	Overlapping toes			Overlapping toes	Overlapping toes	Overlapping toes		
					Arched palate			

a Complete data not available for all patients.

Seven of the eight C112X^Hom^ children were born after uneventful pregnancies and reportedly had a normal perinatal period and initial postnatal development. Patient 5 was born at a gestational age of 30 weeks with initially complicated perinatal adaptation that reportedly resolved without sequelae. All had onset of symptoms within the first year of life (reported age of onset 8 ± 2.7 months), with hypotonia of the paraspinal (truncal) musculature being the first recognized initial symptom in 5/8 patients. A progressive loss of motor abilities, first appearing in the limbs, was noted over the following years, with motor impairment corresponding to level V of the Gross Motor Function Classification System – Expanded and Revised (GMFCS – E&R)^30^ at the time of our evaluations (Supplementary Table 2). All patients exhibited signs of primarily upper motor neuron involvement (UMN) with spasticity both proximally and distally in all four limbs and equally affecting both flexor and extensor musculature. Pathologically brisk deep tendon reflexes with broadened reflex zones were observed in all patients. Seven patients also exhibited Babinski’s sign, while Hoffman’s and Chaddock’s signs were absent, and Oppenheim’s sign was observed unilaterally only in Patient 5 (not tested in Patients 6, 7, and 8). Signs of lower motor neuron involvement (LMN) developed in all patients, with muscular atrophy and fasciculations but strikingly mostly in the truncal axial muscles. Reportedly, all patients initially had normal functions of the bulbar innervated skeletal muscles, but progressive spastic dysarthria and—to a lesser extent—dysphagia appeared beginning in the first year of life. Later, some atrophy of the tongue with fasciculations was observed in 3/8 patients, causing drooling. All identified bulbar symptoms can be attributed to pontine or medulla oblongata-related origin with no clinical indication of mesencephalic lesions. Of note, asymmetrical onset and progression of symptoms and signs in the limbs were observed in the majority of the patients (5/8). All families reported an initial phase of rapid disease progression after the onset of the first symptoms, followed by slowly progressing, mostly spastic motor signs. In 4 of 8 patients from whom detailed information is available, signs of frontal involvement consisting of affective lability with inadequate fits of crying and/or laughter and frontal release signs in the clinical neurological exam were identified (4/8). All patients had orthopaedic issues, such as hip (sub-) luxation and neuromuscular scoliosis. In several patients, recurrent pathological fractures were seen. Functional as well as ultrasound and echocardiographic evaluation as part of routine care did not reveal any structural abnormalities of the visceral organs.

**Figure 1:**
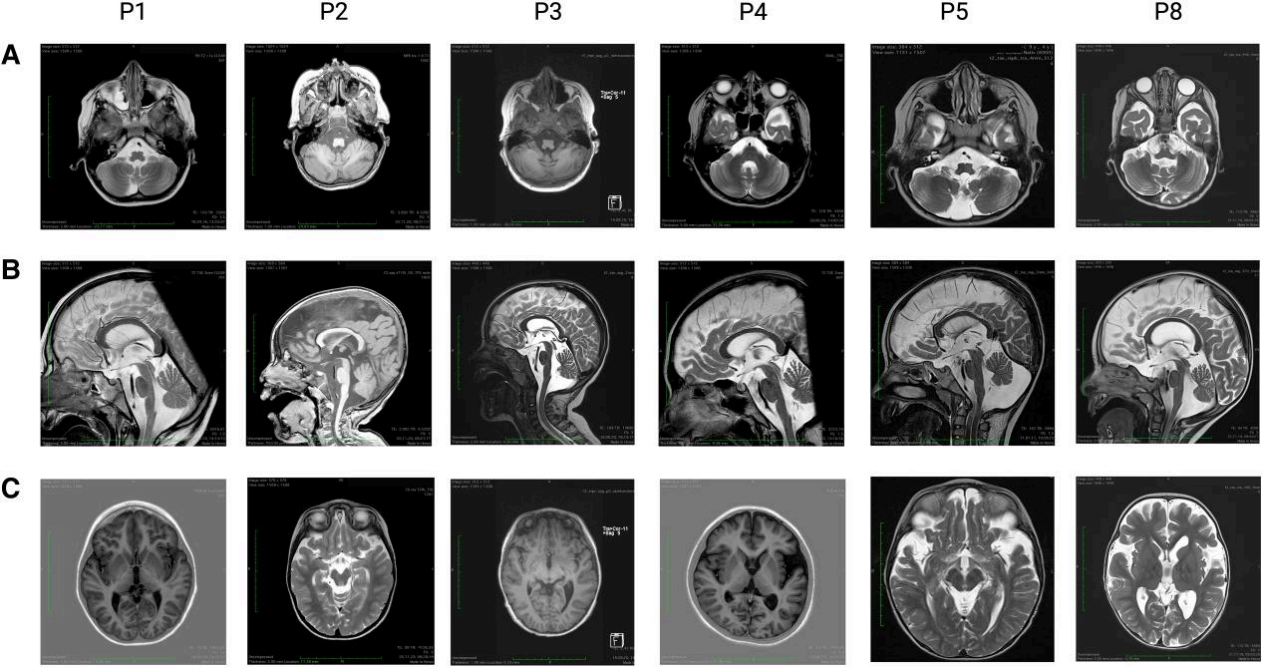
Homozygosity for the p.C112Wfs*11 SOD1 variant (C112X^Hom^) is associated with distinct cranial MRI alterations. Images display coronal and sagittal MRI scans from C112X^Hom^ patients 1, 2, 3, 4, 5, and 8. **(A)** Atrophy of the cerebellar vermis was seen to varying degrees in all analyzed patients. In those individuals who underwent serial imaging studies, the findings progressed over time (Table 2). (**B)** Brain stem atrophy was also observed in all 6 patients. (**C)** In 5/6 patients, some fronto-temporal atrophy was found, which did not progress visibly with age (range of observation period from 9 months to 12 years) in the three patients with serial studies.

All patients underwent cranial MRI at least once, with Patient 1, Patient 2, Patient 4, and Patient 8 having undergone three, four, two, and three examinations, respectively. Imaging data of six patients were available for review in this study. In general, atrophy of specific brain regions was identified in all patients (Fig. 1). Atrophy of the vermis cerebelli was a defining feature and was seen in all studied individuals (6/6). In the four patients who underwent serial imaging, the findings progressed significantly with age. All patients had some brainstem atrophy, which did not show any progression in serial imaging studies except in Patient 8. In 5 of 6 individuals, fronto-temporal cortical atrophy was identified but did not progress significantly over time. In a subset of patients (3/6), thinning of the retro-chiasmal optic tract was identified. A summary of the findings is presented in Table 2.

**Table 2:** Cranial MRI findings in infantile SOD1 deficiency syndrome (ISODDES)

Patient	Age	Cerebellar vermis atrophy	Brainstem atrophy	Fronto-temporal atrophy	Post-chiasmal atrophy	Additional findings
Patient 1	1 year 5 months	–	++	–	–	–
	2 years 5 months	+	++	–	–	–
	5 years 1 month	++	++	–	–	–
Patient 2	9 months	+	++	+++	–	–
	1 year 4 months	++	++	+++	–	Normal MR spectroscopy
	2 years 4 months	+++	++	+++	+	–
	4 years	+++	++	+++	+	Normal MR spectroscopy
Patient 3	5 years 1 month	+++	+	+++	–	Periventricular FLAIR hyperintensity within the corona radiata mega cisterna magna
Patient 4	4 years 6 months	+++	++	+	+	–
Patient 5	9 years	+++	++	++	–	–
	12 years 1 month	+++	++	++	–	–
Patient 8	1 year 2 months	+	+	+++	+	–
	2 years 2 months	+++	++	+++	+	Normal MR spectroscopy
	2 years 5 months	+++	++	+++	+	–

**Figure 2:**
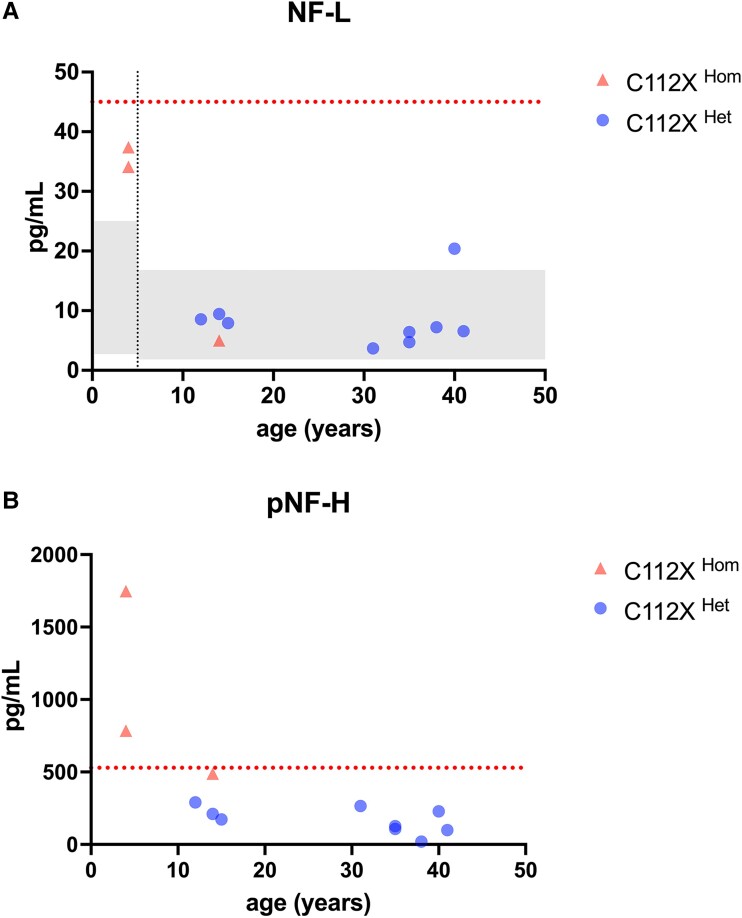
**Plasma levels of neurofilament light and phosphorylated neurofilament heavy chain are elevated in younger SOD1-deficient individuals graphs depict plasma levels of neurofilament light (NF-L) and phosphorylated heavy chain (pNF-H) levels in samples from patients 2, 3, and 4, plus nine heterozygous individuals.** Values were plotted against age to visualize potential age-related differences. (**A**) Younger patients exhibited levels of NF-L above the range observed in healthy, age-matched controls (0–4 years, left grey square), while the older patient 4 had levels within the range observed in controls of the same age range (5–18 years, right grey square). Reference ranges were previously reported by Nitz *et al*.^31^ P-NfL levels were below the cut-off for adult-onset ALS in all samples.^24^ (**B**) pNF-H levels were elevated and above the cut-off used in ALS patients in both younger patients (patients 2 and 3), while the older patient 4 showed levels just below. All heterozygous individuals exhibited non-elevated pNF-H levels.

Analysis of neurofilament light chain (NF-L) and phosphorylated neurofilament heavy chain (pNF-H) in plasma was performed to assess ongoing neuroaxonal damage in patients 2, 3, and 4 and asymptomatic heterozygous relatives (the parents of three patients and the siblings of Patient 3) (Fig. 2). In the C112X^Hom^ patients, we observed higher levels of both markers in the two patients younger than 5 years of age that were above the range observed in healthy children of that age group in several previous independent studies (age 0–4 years: median 7.12 pg/mL, range 2.73–25 pg/mL^31^ and median 10.0 pg/mL, IQR 7.2–12.4 pg/mL^32^). In contrast, Patient 4 (sample collected at 13 years of age) exhibited levels within the range observed in healthy children aged 5–18 years (median 4.07 pg/mL, range 1.84–16.8 pg/mL)^31^ and similar to those seen in asymptomatic adult relatives. The younger patients had pNF-H levels above the cut-off for adult-onset ALS patients (cut-off: 529 pg/mL; Patient 2: 1748, Patient 3: 785 pg/mL), while NF-L levels were below the cut-off of 45 pg/mL (Patient 2: 37.4 pg/mL, Patient 3: 34.1 pg/mL). The heterozygous relatives had levels within the reference ranges for both markers. Due to the limited number of patients analysed, no formal statistical evaluation of measured NF levels was performed.

### The *SOD1* variant c.335dupG encodes a truncated, non-functional unstable protein with low aggregation propensity

**Figure 3:**
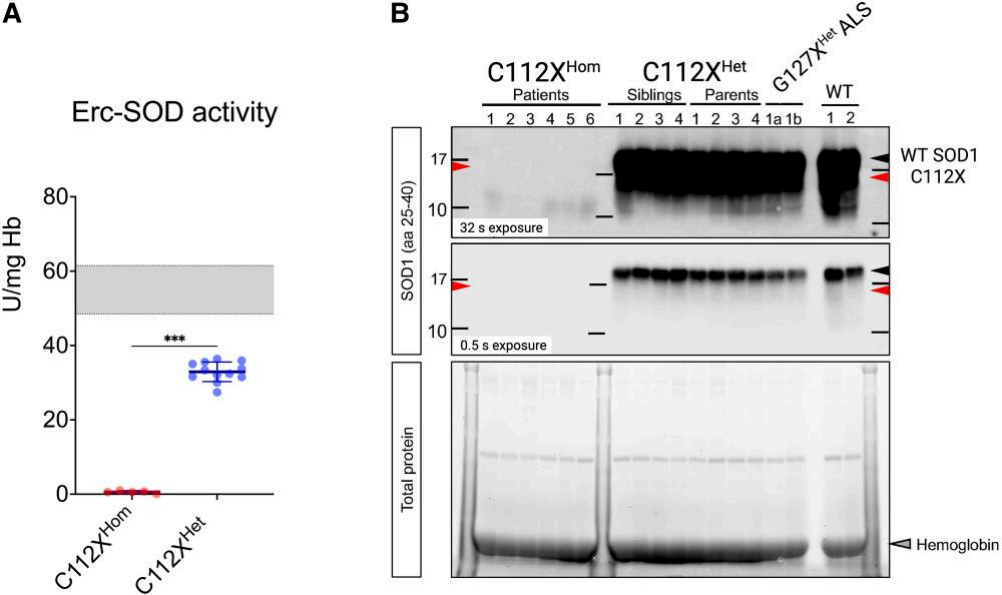
**Absence of SOD activity and SOD1 protein in erythrocyte lysates from homozygous individuals (A)** Erythrocyte samples from patients homozygous for the *SOD1* c.335dupG, p.C112Wfs*11 (C112X^Hom^) are deficient in SOD1 enzyme activity (0.6 ±0.4 U/mg Hb, *P* < 0.001 *n* = 8). In samples from heterozygous carriers (C112X^Het^), the enzyme activity was approximately halved (32.9 ± 2.6, U/mg Hb, *n* = 12) compared to WT control samples (reference range 48.5-61.5 U/mg Hb, indicated by the grey box). Data are presented as the mean ± SD and were analyzed using the Mann-Whitney U test. *** *P* < 0.001. (**B)** Immunoblotting of erythrocyte lysates using an antibody raised against amino acid residues 25-40 of SOD1 showed positive detection of WT-SOD1 but not p.C112X in heterozygous carriers (*n* = 8). No SOD1 protein was detected in the samples from homozygous individuals (P1-P6, *n* = 6) even after extended exposure time (upper panel). A previous study on the lysates of proteasome-inhibited fibroblasts identified a 13-kDa truncated SOD1 protein (arrow C112X) as a product of *SOD1* c.335dupG, p.C112X. In addition, WT- SOD1 (black arrow), but not the unstable truncated mutant protein was readily detected in lysates from individuals heterozygous for *SOD1* G127X (also known as p.K128Gfs*6, G127X^Het^), a truncating mutation previously demonstrated to cause adult-onset familial ALS (n = 2). See Supplementary Material for uncropped blots.

SOD1 enzyme activity was virtually absent in erythrocyte lysates in all six studied homozygous patients, confirming the results from previous studies.^21,22^ Heterozygous carriers exhibited approximately halved SOD1 activity when compared to the reference range derived from healthy controls (Fig. 3A). We used western blotting to investigate the presence of the mutant protein in haemolysates from homozygous and heterozygous carriers of C112X. Neither wild-type (WT) nor truncated SOD1 was detected in haemolysates from homozygous individuals, while only WT SOD1 was identified in samples from heterozygous carriers, indicating low stability and efficient degradation of the truncated p.C112X SOD1 (Fig. 3B). Full images of the blots and the total protein load are available in the Supplementary material. Previous research has indicated that mutated mRNA is present both in homozygous and heterozygous carriers of C112X, arguing against non-sense mediated RNA decay.^21^

**Figure 4:**
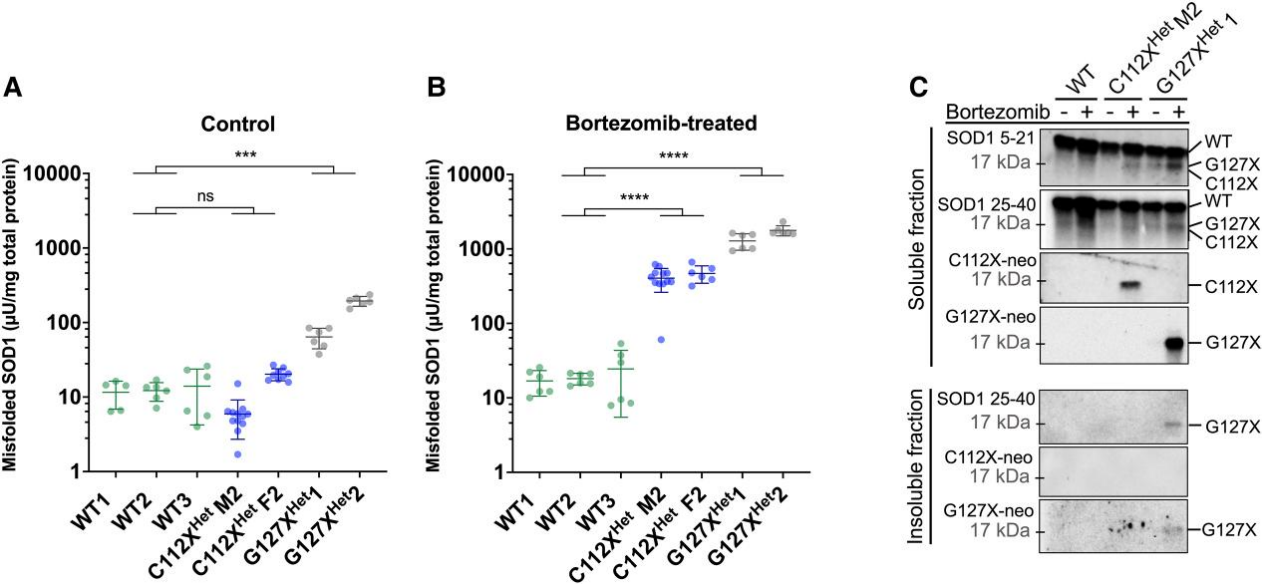
**The truncated SOD1 C112X is efficiently degraded and exhibits low aggregation propensity.** (**A, B**) Plots show levels of soluble misfolded SOD1 detected with an ELISA that specifically detects misfolded, but not natively folded, SOD1 (misELISA) in fibroblast culture extracts from control (WT) (*n* = 3), C112X^Het^ carriers- (*n* = 2), or G127X^Het^ ALS patients- (*n* = 2). All fibroblast lines were derived in house from skin biopsies. **(A)** Cells from C112X^Het^ individuals show levels of misfolded SOD1 similar to those in control cells under standard culture conditions (*P* > 0.9999), whereas misSOD1 levels are significantly increased in fibroblasts carrying the previously described ALS-causing truncated variant p.G127X (*P* = 0.0006). **(B)** Proteasome inhibition using bortezomib (5 ng/mL) resulted in significantly increased levels of misSOD1, both in cells from C112X^Het^ carriers (*P* < 0.0001) and G127X^Het^ ALS patients (*P* < 0.0001) when compared to WT cells. Data are expressed as the mean ± SD of four to twelve technical replicates from 2–3 independent experiments. The means were compared using the Kruskal–Wallis test followed by Dunn’s multiple comparisons test. *P < 0.05, **P < 0.01, ***P < 0.001, **** *P* < 0.0001, ns, not significant. **(C)** Western blot on fibroblast extracts. Both WT SOD1 and the truncation variants p.C112X and p.G127X were detected in the soluble fraction using antibodies against the *N*-terminal sequences of SOD1 (SOD1 5–21 and SOD1 25–40 aa, respectively) and antibodies raised against the p.C112X, and the p.G127X neo-peptides (C112X-neo and G127X-neo, respectively). The p.G127X but no p.C112X was detected in the insoluble fraction. aa—amino acid, WT—wild-type, C112X^Het^ M2—mother of Patient 2, C112X^Het^ F2—father of Patient 2. The migration pattern varies between the WT and the different truncation variants, as indicated on the right. See Supplementary material for uncropped blots.

In previous studies we found that primary fibroblast cells derived from homozygous patients were extremely sensitive to ambient oxygen concentrations,^21^ and we were unable to establish fibroblast cell lines from the sparse primary cells growing out from the patient-derived skin biopsies. Therefore, we established fibroblast cell lines from heterozygous C112X (C112X^Het^) carriers and used these as the basis for further studies of p.C112X. As the mutant protein is severely truncated and missing the C112 residue, it can neither adapt the normal folding nor form the stabilizing C7-C112 disulfide bond. Thus, any mutant protein in the cells should exist as a disordered or misfolded variant. Quantification using an ELISA that specifically detects misfolded SOD1 (misELISA) detected no significant increase in misfolded SOD1 in the C112X^Het^ fibroblasts in comparison to WT controls under normal culture conditions (Fig. 4A). In contrast, cultured fibroblasts derived from ALS patients heterozygous for the ALS-causing truncating variant p.G127X (also known as p.K128Gfs*6) (G127X^Het^) had significantly increased levels of misfolded SOD1. Culturing in the presence of the proteasome inhibitor bortezomib resulted in a minor increase in soluble misfolded SOD1 in the controls, whereas a significant increase was detected in both C112X^Het^ and G127X^Het^ fibroblasts (Fig. 4B). We also analysed both soluble and insoluble fractions using western blot (WB) and detected low levels of the p.C112X variant after proteasome inhibition in the soluble fraction. Fractions of the p.G127X truncation variants were also detected in the insoluble fraction. These results were consistent with previous findings.^26^ In contrast, we were unable to detect aggregated SOD1 in the insoluble fraction from the proteasome-inhibited p.C112X fibroblasts (Fig. 4C). Taken together, these findings suggest that the p. C112X variant has low stability, is efficiently cleared by proteasomal degradation, and has low aggregation propensity in skin-derived fibroblasts.

### Loss of SOD1 activity has limited effects on non-neuronal organ function

**Figure 5:**
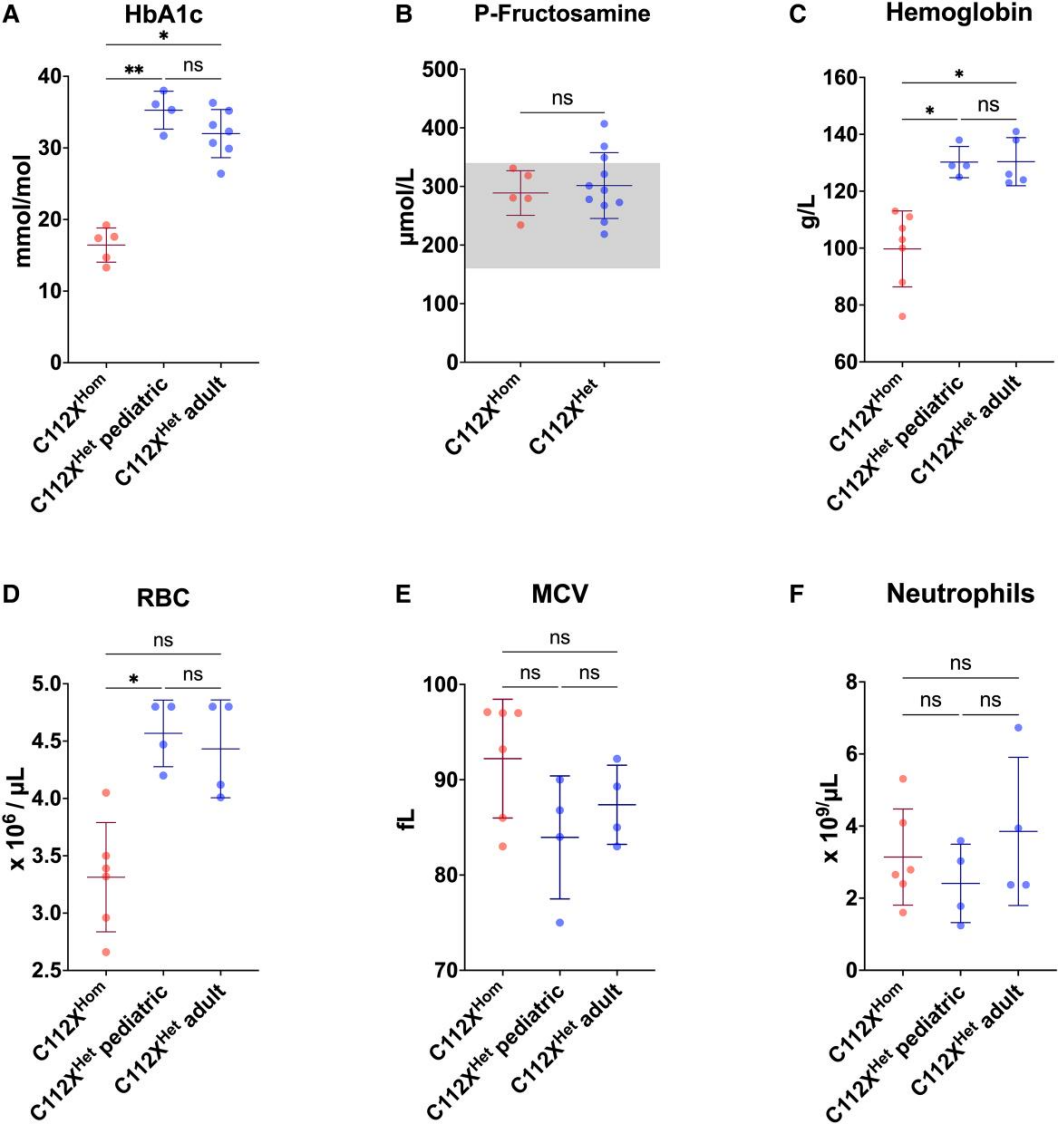
**Homozygosity for *SOD1* C112X results in anaemia with low haemoglobin, reduced red blood cell counts, and shortened lifespan.** Patients homozygous for the C112X variant (C112X^Hom^) show a haematological phenotype with reduced haemoglobin (Hb) **(C)** and red blood cell (RBC) **(D)** counts when compared to age-matched heterozygous carriers (C112X^Het^ paediatric) and heterozygous adults (C112X^Het^ adult). The mean corpuscular volume (MCV) was increased in homozygous patients but did not reach statistical significance. Significantly decreased HbA1c values indicated decreased erythrocyte lifespans. Plasma (P) fructosamine levels were within reference ranges (box) with no statistically significant difference between C112X^Hom^ and C112X^Het^ carriers, indicating non-diabetic metabolic state **(B)**. No significant difference was seen between levels of neutrophils (homozygous: *n* = 6 for RBC **(D)**, MCV **(E)**, and neutrophils **(F)**, *n* = 7 for haemoglobin, and *n* = 5 for HbA1c **(A)** and fructosamine **(B)**, heterozygous children: *n* = 4, heterozygous adults: *n* = 4 for RBC, MCV, and neutrophils, *n* = 7 for HbA1c, and *n* = 11 for fructosamine). Data presented as mean ± SD. Means were compared using Mann–Whitney U or Kruskal–Wallis tests, followed by followed by Dunn’s multiple comparisons test. * *P* < 0.05, ** *P* < 0.01, *** *P* < 0.001.

SOD1 is ubiquitously expressed, and normal SOD1 enzyme activities are six-fold and three-fold higher in the liver and kidney, respectively, than in the CNS.^33,34^ Therefore, we hypothesized that peripheral organ function might also be affected by SOD1 deficiency. To examine this in detail, we assessed a comprehensive panel of clinically established markers of organ dysfunction in our cohort of homo- and heterozygous individuals (Supplementary Table 3). We found no evidence for liver and kidney dysfunction and only modest deviations in some of the other variables measured. The homozygous patients had significantly reduced levels of plasma (P-) creatinine (25.0 ± 5.8 µmol/L, *P* < 0.001) when compared with heterozygous carriers (56.2 ± 14.8 µmol/L). This was most likely because of the loss of skeletal muscle tissue and not hyperfiltration since the levels of the glomerular filtration rate marker P-cystatin C were normal. However, there was no increase in the muscle damage marker P-creatine kinase (CK) (Supplementary Table 3). P-insulin levels were significantly reduced in homozygous individuals when compared with heterozygous carriers (5.5 ± 1.7 mIU/L versus 17.2 ± 14.9 mIU/L, *P* = 0.001), as was P-C-peptide, albeit still within reference ranges (0.49 ± 0.05 nmol/L and 1.06 ± 0.44 nmol/L, respectively, *P* < 0.001). To further assess whether these changes lead to a diabetic metabolic state, we analysed the glycated haemoglobin (HbA1c) levels in erythrocytes (Fig. 5A). Despite decreased insulin levels, the HbA1c levels were below the reference ranges in the six homozygous patients (16.4 ± 2.4 mmol/mol, reference range 27–42 mmol/mol) and were significantly reduced when compared with heterozygous carriers (33.2 ± 2.4 mmol/mol, *P* < 0.001), indicative of a high erythrocyte turnover. We further studied plasma fructosamine (P-fructosamine) as an alternative measure of protein glycation and found no significant difference between C112X^Hom^ individuals (288.8 ± 38.1 µmol/L) and heterozygous carriers (301.5 ± 56.2 µmol/L, *P* = 0.91, reference range 160–340 µmol/L, Fig. 5B).^35^ Frequent measurements of fasting and postprandial blood glucose levels showed no elevation above normal ranges in any of the patients. P-ACTH was reduced in homozygous patients, whereas P-fT4 and P-PO_4_ levels were elevated but within reference ranges when corrected for age. In addition, P-ALAT levels were elevated slightly above reference ranges in C112X^Hom^ individuals without reaching statistical significance (*P* = 0.57).

### Patients lacking SOD1 activity exhibit a haematological phenotype characterized by anaemia and shortened red blood cell survival

To study the effect of a loss of SOD1 function on the haematopoietic system, complete blood counts were assessed in homo- and heterozygous individuals (Fig. 5, Supplementary Table 4). Haemoglobin levels were significantly reduced, indicating anaemia (reference 107–139 g/L) in homozygous children (99.7 ± 13.3 g/L) when compared with age-matched heterozygous carriers (130 ± 5.5 g/L, *P* = 0.01) and adult heterozygous relatives (130 ± 8.4 g/L, *P* = 0.02, Fig. 5C). Similarly, homozygous individuals had significantly reduced red blood cell counts (RBCs) compared to age-matched heterozygous carriers (heterozygous: 4.57 ± 0.35 × 10^6^/µL, homozygous: 3.31 ± 0.48 × 10^6^/µL, *P* = 0.02), while the difference compared with adult heterozygous individuals was not statistically significant (4.43 ± 0.43 × 10^6^/µL, *P* = 0.07, Fig. 5D). While the mean corpuscular volume (MCV) was increased above the reference ranges in 4/8 of the homozygous patients, no statistically significant difference was found compared with age-matched heterozygous individuals (92.22 ± 6.23 fL versus 83.95 ± 6. 45 fL, *P* = 0.28) or adult heterozygous carriers (87.38 ± 4.15 fL, *P* = 0.63, Fig. 5E). Reticulocytes were intermittently elevated in samples from patients 1, 2, and 4 (no data available for other patients, Supplementary Table 4). Normal P-haptoglobin and P-bilirubin values close to the upper reference limit also indicated a moderate degree of haemolysis (Supplementary Table 3).

To assess whether altered RBC variables in homozygous individuals were associated with shortened RBC survival, we used the HbA1c values to estimate mean erythrocyte age (Fig. 5A).^36^ The low mean HbA1c level of the patients indicated approximately halved erythrocyte lifespans. There were no alterations in bone marrow-derived leukocytes (Fig. 5F, Supplementary Table 4), suggesting that the effects of the loss of SOD1 activity are specific for the erythropoietic lineage.

### Loss of SOD1 activity is characterized by specific alterations in glutathione metabolism

**Figure 6:**
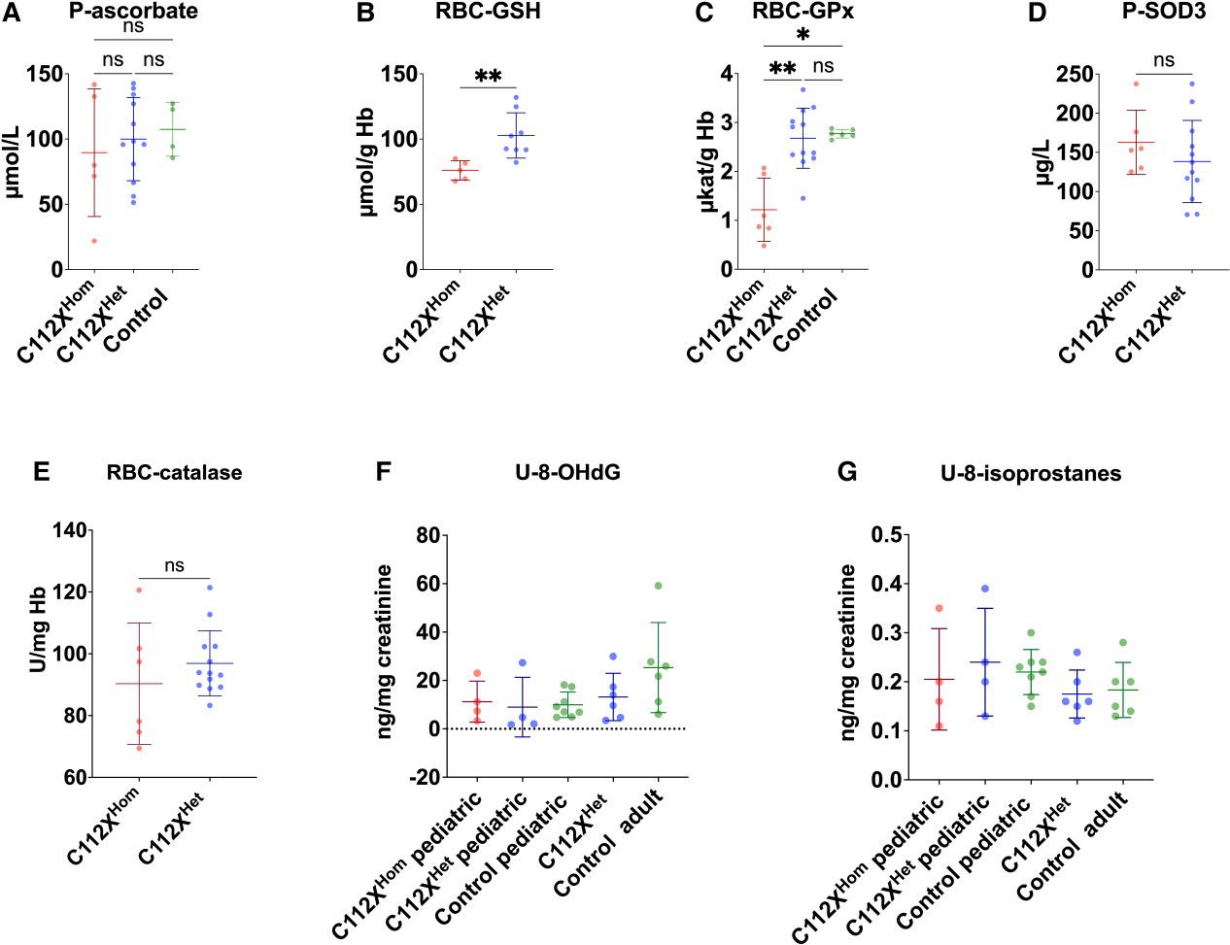
**SOD1 deficiency leads to altered glutathione metabolism, while commonly used markers for oxidative stress remain unaltered.** (**A**) Despite high reactivity with superoxide, plasma ascorbate (P-ascorbate) levels were not significantly different between homozygous (C112X^Hom^) and heterozygous individuals (C112X^Het^) or controls. C112X^Hom^*n* = 5, C112X^Het^*n* = 12, control *n* = 4. **(B)** In contrast, whole blood reduced glutathione (RBC-GSH) levels were significantly reduced in homozygous individuals (76 ± 7.4 µmol/g Hb, *P* = 0.002) when compared to heterozygous carriers (102.8 ± 17.4 µmol/g Hb). C112X^Hom^*n* = 5, C112X^Het^*n* = 8. **(C)** Levels of the H_2_O_2_-metabolizing GSH-utilizing glutathione peroxidase-1 (RBC-GPx) were significantly reduced in homozygous individuals (1.2 ± 0.6 µkat/g Hb) when compared to both heterozygous carriers (2.7 ± 0.6 µkat/g Hb, *P* = 0.003) and controls (2.8 ± 0.1 µkat/g Hb, *P* = 0.01). C112X^Hom^*n* = 6, C112X^Het^*n* = 12, control *n* = 6. **(D)** The levels of extracellular SOD3 were not significantly different between homozygous (162.8 ± 41 µg/L) or heterozygous individuals (138.4 ± 52.5 µg/L) (*P* = 0.26, reference range 142 ± 43 µg/L). C112X^Hom^*n* = 6, C112X^Het^*n* = 12. **(E)** No significant difference In the activity of the H_2_O_2_-metabolizing RBC-catalase was identified between homozygous patients and heterozygous carriers (90.3 ± 19.6 U/mg Hb and 96.9 ± 10.5 U/mg Hb, respectively, *P* = 0.43). C112X^Hom^*n* = 6, C112X^Het^*n* = 12. **(F, G)** Despite distinct alterations in redox metabolism, no overt differences were seen in the urinary excretion of 8-hydroxydeoxyguanosine (U-8-OHdG), a marker of oxidative damage to DNA, and urinary (U-)8-isoprostanes, a marker of lipid peroxidation. C112X^Hom^*n* = 4, C112X^Het^ paediatric *n* = 4, C112X^Het^ adult *n* = 6, control paediatric *n* = 8, control adult *n* = 6. Data were compared using the Mann–Whitney U test (A-E) or Kruskal–Wallis test (F, G). *P < 0.05, **P < 0.01, ns: not significant.

We next determined the levels of antioxidant enzymes and low-molecular-weight compounds as well as markers of oxidative damage to characterize the effects of the loss of SOD1 on redox metabolism. Despite the high reactivity of ascorbate with superoxide,^37^ plasma ascorbate levels were not significantly different between homozygous and heterozygous individuals or WT controls (homozygous: 89.6 ± 48.9 µmol/L, heterozygous: 99.9 ± 31.9 µmol/L, controls: 107.4 ± 20. 6 µmol/L, Fig. 6A). However, homozygous individuals exhibited significantly decreased levels of reduced glutathione in erythrocytes (RBC-GSH) (Fig. 6B). In addition, the activity of the GSH-utilizing enzyme glutathione peroxidase-1 in erythrocytes (RBC-GPx, Fig. 6C), which catalyses the reduction of hydrogen peroxide (H_2_O_2_), was significantly reduced in homozygous individuals compared to both heterozygous carriers and controls. In contrast, no significant differences in plasma SOD3 levels were detected in homozygous (162.8 ± 41 µg/L) or heterozygous individuals (138.4 ± 52.5 µg/L) (Fig. 6D, reference range 142 ± 43 µg/L^38^). The activity of the H_2_O_2_-metabolizing catalase in erythrocytes (RBC-catalase) was not significantly different between homo- and heterozygous carriers (Fig. 6E). Analysis of urinary excretion of 8-OHdG, a marker of oxidative damage to DNA, and 8-isoprostanes, a marker of lipid peroxidation, revealed no significant differences between groups (Fig. 6F and G).

## Discussion

The crucial role of SOD1 among antioxidant detoxification mechanisms is well established. We previously found that skin fibroblasts from a C112X^Hom^ patient were extremely sensitive to molecular oxygen. Only when the oxygen tension was reduced to 2% and a protective ascorbate derivative was added to the medium, did a narrow halo of slowly growing dysmorphic fibroblasts appear around the skin biopsies.^21^ Moreover, in a knockdown screen of thousands of genes, human iPSC-derived neuronal cells were found to be exceptionally vulnerable to reductions in SOD1.^39^ Thus, the discovery of the homozygous C112X variant as a loss-of-function variant in humans raises intriguing questions regarding the general consequences of a loss of SOD1 function in the body. In our cohort of eight homozygous children, SOD1 activity was confirmed to be absent (Fig. 3). As reported previously, heterozygous individuals have an approximately halved enzyme activity.^21,22^ All homozygous individuals in this study showed the same uniform and severe neurological phenotype, while none of the heterozygous carriers showed any neuromuscular or neurodegenerative symptoms during our four-year observation period. We conclude that a ≈50% reduction in SOD1 enzyme activity and the presence of a single C112X *SOD1* allele is tolerated, at least up to age 45 years.

Despite the previously demonstrated detrimental effects of a loss of SOD1 function in cultured cells and the high expression of SOD1 in virtually all organ systems, we found little evidence of visceral organ damage in any of the affected children (Supplementary Table 3). The biopsy sites for fibroblast cultures and other wounds reportedly healed normally, and no dermal phenotype was observed. Indirect evidence of muscular wasting and disturbed pancreatic islet cell function was found. However, the observed values for the markers of islet cell function were within age-adapted reference ranges, and frequent spontaneous glucose measurements did not reveal any episodes of hyperglycaemia. These findings indicate that the changes in islet cell physiology are currently subclinical. The relevance of the observed differences in ACTH, fT4, and phosphate levels (Supplementary Table 3) between homozygous and heterozygous individuals remains to be elucidated, although these were also within the reference ranges for both groups. In general, the apparent contrast between findings from cell culture and our observations in SOD1-deficient individuals might reflect the incomplete reproduction of redox-associated pathophysiology in 2D cell culture models.^40,41^

Given the surprisingly well-preserved organ function in SOD1 deficiency, we hypothesized that alternative detoxification processes might compensate for the loss of antioxidant defence. Evidence from mouse knockout models suggest that there is no or only limited functional complementation between the three SOD isoenzymes.^42,43^ This might be explained by the fact that the substrate, the superoxide anion radical, is charged and therefore penetrates membranes poorly. Ascorbate has a high reactivity with superoxide and should be the most important scavenger of the radical in the cytosol after SOD1. It is, unlike in mice, not synthesized in humans.^37,44^ However, ascorbate levels in plasma were not significantly different between groups but showed large interindividual differences among the C112X^Hom^ patients (Fig. 6A). Thus, dietary intake and efficient regeneration of oxidized ascorbate seem to suffice to keep the levels normal. In contrast, we observed distinct alterations in glutathione metabolism in individuals homozygous for the *SOD1* C112X variant, all of whom showed a significant reduction in reduced glutathione. This antioxidant tripeptide plays a major role in the redox defense of the CNS,^45^ but is also highly expressed in organs such as the liver and kidney,^46^ for which we detected no overt abnormalities when assessing parameters of organ function. However, the decreased GSH contents might have been caused by the prooxidant effects of haemoglobin and may thus be limited to erythrocytes.^47^ No differences in GSSG levels or the GSH/GSSG ratio were found (data not shown). However, since some artificial oxidation generating GSSG will inevitably occur during the preparation of samples,^48^ no conclusions with regards to changes in GSSG levels can be drawn. Specific analysis of GSSG requires derivatization of GSH at the time of sampling, which could not be accomplished in this study.^48^ Reduced glutathione (GSH) is known to react with superoxide,^49^ although this reaction is relatively slow in comparison to other radical reactions^50^ with questionable relevance in the physiological state, i.e. the presence of superoxide dismutases. Nonetheless, oxidation of GSH by superoxide might occur at significant levels in the context of reduced or absent SOD activity.^51^

Similar to the phenotype in genetic disorders associated with GSH deficiency,^52,53^ we observed significantly reduced haemoglobin levels in conjunction with decreased RBC counts (Fig. 5) as well as reduced erythrocyte survival estimated by decreased HbA1c levels. Furthermore, the observed changes in RBC survival (Fig. 5) are in line with findings from murine SOD1 KO models, where anaemia with decreased RBC lifespan and increased average erythrocyte sizes were accompanied by significantly decreased RBC-GPx activity and protein levels.^54^ From a therapeutic perspective, treatment with the GSH precursor *N*-acetylcysteine^55^ improved anaemic symptoms,^54^ as well as other symptoms in SOD1 knockout models^56^ and might be a promising approach to treat symptoms observed in the patients studied here. The activity of RBC-GPx, which was significantly reduced in SOD1-deficient patients, has previously been demonstrated to correlate positively with SOD1 enzymatic activity and is increased in individuals with trisomy 21.^57^ The mechanism behind this correlation is incompletely understood, but it has been shown that superoxide can directly inactivate RBC-GPx in vitro and that GSH can reverse this effect.^58^ Taken together, these findings indicate that the loss of SOD1 function primarily affects glutathione metabolism with no distinguishable effect on ascorbate homeostasis. Interestingly, recent studies have also suggested reduced GSH levels in ALS.^59–61^ A generally sufficient compensation at the organism level might explain the contrast between normal levels of the commonly used oxidative stress markers urinary 8-OHdG and 8-isoprostanes and the tissue-specific damage indicated by motor system impairment and the haematological phenotype. Tissue-specific analysis of markers of oxidative damage might allow a more detailed assessment of end organ damage.

The identification of p.C112X-associated loss of SOD1 function resulting in a human phenotype caused renewed interest in the downstream consequences in the context of ALS and beyond. Recently, de Souza *et al*. reported the same severe phenotype in C112X^Hom^ children of Lebanese origin.^62^ Supporting that the phenotype in C112X^Hom^ children is caused by the absence of SOD1 enzyme activity is the discovery of a similar primarily neurological phenotype in an infant homozygous for another *SOD1* mutation c.357_357 + 2delGGT. This mutation results in the omission of either valine at position 119 or 120 of SOD1, and similar to the cases described here, neither SOD1 enzymatic activity nor mutant SOD1 protein was detected in erythrocyte lysates, again arguing that a loss rather than the gain of a toxic function is a principal component of the pathomechanism.^25^ In addition to this new human case, a truncating homozygous loss-of-function mutation in *SOD1* was recently identified in Markiesje dogs that exhibited progressive tetraparesis and muscle wasting, in addition to brainstem and spinal cord atrophy.^63^ However, it is notable that *Sod1* knockout mice do not replicate the severe motor neuron phenotype that develops in humans despite developing a significant, motor neuropathy in later life in addition to visceral symptoms such as liver tumours, sarcopenia, and ocular symptoms.^56,64–66^ Given the relatively young age of the patients described here, later-onset conditions associated with a loss of SOD1 function cannot be ruled out at this stage.

Our analyses of the mutant p.C112X SOD1 further support the notion of a loss of enzyme activity as a major driver of the observed phenotype. Using a previously established ELISA with high sensitivity and specificity to disordered SOD1 and immunoblotting of aggregated SOD1 in the insoluble fraction of fibroblast lysates, we found both low aggregation propensity and efficient proteasomal degradation of the mutant protein (Fig. 4). This is in contrast to the currently known ALS-mediating SOD1 mutants, which exhibit an increased inherent aggregation propensity.^14^ Taken together, these findings suggest that homozygosity for loss-of-function variants in *SOD1* results primarily in an infantile-onset motor neuron condition but with additional neuronal and non-neuronal involvement, which distinguishes it from typical adult-onset ALS. We propose the term ‘infantile superoxide dismutase 1 deficiency syndrome’ (ISODDES). A comparison of ISODDES with adult-onset ALS caused by homozygosity for the p.D91A SOD1—which has normal enzymatic activity—and the truncated p.G127X SOD1, causing ALS in heterozygosity and has no enzymatic activity, is presented in Supplementary Table 5.

SOD1 is generally regarded as an antioxidant enzyme that protects biomolecules against the direct and indirect toxic effects of superoxide by dismutating the radical to hydrogen peroxide. However, the enzyme has also been claimed to exert ‘non-canonical’ functions not related to oxidant injury. In yeast, SOD1 has been found to form complexes with casein kinases and to stabilize these by localized formation of hydrogen peroxide. This pathway can transduce the repression of respiration by oxygen and glucose.^67^ In a mammalian cell line, a partly analogous mechanism involving the stabilization of casein kinase 1-y has been suggested to be essential for the Wnt signalling pathway.^68^ Moreover, oxidative stress has been found to lead to phosphorylation of serines in SOD1 in complex with the effector kinase Dun1 in yeast.^69^ After translocation to the nucleus, phosphorylated SOD1 binds to promoters regulating the expression of oxidative resistance and repair genes. The existence of this pathway has not been confirmed in mammalian cells, but nuclear and nucleolar SOD1 has been reported to be important for ribosomal subunit maturation.^70^ The reportedly normal development in utero and in early infancy of C112X^Hom^ patients suggests that, in those phases, SOD1 deficiency does not cause any significant perturbations in Wnt signalling, ribosomal maturation or in signalling by the superoxide radical. However, it cannot be excluded that perturbed ‘non-canonical’ SOD1 functions or signalling by the superoxide radical might play a role in the later developing motor neuron dysfunction.

The concentration of the ubiquitously expressed SOD1 in motor areas is moderate and not different from that in other parts of the CNS.^34^ Nevertheless, our results indicate that of all the organs and functions in the human body, the absence of SOD1 has the greatest detrimental effect on the CNS and, in particular, the motor system. Similarly, ALS-causing mutations in *SOD1* (some 220 mutations have been found globally) exert damage specifically in the motor centres of the CNS, although by a gain-of-function mediated by the formation of mutant protein aggregates with a prion-like function.^15,16^ No significant aggregation or injury to other parts of the body has been detected. This specific vulnerability of the motor system to the different perturbations in the ubiquitously expressed SOD1 is remarkable and enigmatic. We have previously shown that misfolded SOD1 species are enriched in motor areas compared to other parts of the CNS and peripheral organs,^71–73^ as well as in iPSC-derived motor neuron cultures.^74^ Moreover, inclusions containing misfolded SOD1 regularly appear in motor neurons of sporadic ALS patients and patients with mutations in ALS-causing genes other than *SOD1*.^75–79^ Altered maturation and modifications in SOD1 have also been demonstrated in such patients.^80^ It is currently not known whether these aberrations in SOD1 have any pathogenic role.^81^ Hypothetically, the ISODDES findings presented here combined with almost 30 years of research into how mutations in a ubiquitously expressed protein cause adult-onset ALS are best explained by SOD1 having one or more yet-to-be-defined specific roles in motor areas. These non-canonical functions might cause altered metabolism of SOD1, resulting in an increased population of misfolded aggregation-prone species in the motor system, thus explaining the motor system-specific aggregation seen in SOD1-ALS. Any perturbation of SOD1 homeostasis would accordingly first and foremost be manifested by symptoms from the motor system, as observed in adults with ALS heterozygous for *SOD1* mutations and in children homozygous for C112X and V120delV.

The association of the severe ISODDES phenotype with a loss of SOD1 function suggests the need for cautious application of general silencing interventions in ALS since the currently studied gene therapy approaches equally target the mutant and wild-type SOD1 alleles. However, given the absence of an overt phenotype in heterozygous carriers with approximately halved enzyme activity, it is possible that there is a threshold of minimum SOD1 activity to avoid disease manifestation. Further evaluation of the relation between a loss of SOD1 function and human disease is essential for the application of SOD1 modulation therapies in ALS.

## Supplementary Material

fcad017_Supplementary_Data

## Abbreviations

ALS =: amyotrophic lateral sclerosis
CK =: creatine kinase
DTPA =: diethylenetriaminepentaacetic acid
GMFCS =: gross motor function classification system
GPX =: glutathione peroxidase
GSH =: reduced glutathione
GSSG =: oxidized glutathione
Hb =: haemoglobin
HbA1c =: glycated haemoglobin
HPLC =: high performance liquid chromatography
iPSC =: induced pluripotent stem cells
ISODDES =: infantile superoxide dismutase 1 deficiency syndrome
LMN =: lower motor neuron
MCV =: mean corpuscular volume
misELISA =: disordered SOD1-specific enzyme-linked immunosorbent assay
MPA =: meta-phosphoric acid
NF-L =: neurofilament light chain
pNF-H =: phosphorylated neurofilament heavy chain
RBC =: red blood cell
ROS =: reactive oxygen species
SARS-CoV2 =: Severe acute respiratory syndrome coronavirus type 2
SOD1 =: superoxide dismutase 1
UMN =: upper motor neuron
WB =: western blot
WT =: wild-type
8-OHdG =: 8-hydroxy-2’-deoxyguanosine
