# Supplementary material for: The motor system is exceptionally vulnerable to absence of the ubiquitously expressed superoxide dismutase-1
Source: Brain Commun. 2023 Jan 27;5(1):fcad017. doi: 10.1093/braincomms/fcad017 (PMC9924500; doi:10.1093/braincomms/fcad017)
Supplement: fcad017_Supplementary_Data [file fcad017_supplementary_data.pdf]

SUPPLEMENTARY MATERIAL

The motor system is exceptionally vulnerable to absence of the ubiquitously expressed superoxide dismutase-1

Julien H. Park,[1,2] Ulrika Nordström,[1] Konstantinos Tsiakas,[3] Isil Keskin,[4] Christiane Elpers,[2] Manoj Mannil,[5] Raoul Heller,[6] Melinda Nolan,[6] Salam Alburaiky,[6] Per Zetterström,[7] Maja Hempel,[3,8] Ulrike Schara,[9] Saskia Biskup,[10] Petra Steinacker,[11] Markus Otto,[11] Jochen Weishaupt,[12] Andreas Hahn,[13] René Santer,[3] Thorsten Marquardt,[2] Stefan L. Marklund,[7] and Peter M. Andersen[1]

Author affiliations:

[1] Department of Clinical Sciences, Neurosciences, Umeå University, 901 87, Umeå, Sweden.

[2] Department of General Paediatrics, University of Münster, 48149 Münster, Germany.

[3] Department of Paediatrics, University Medical Centre Hamburg-Eppendorf, 20251 Hamburg, Germany.

[4] Department of Medical Biosciences, Pathology, Umeå University, 901 87, Umeå, Sweden.

[5] Clinic for Radiology, University Hospital Münster, WWU University of Münster, 48149 Münster, Germany.

[6] Starship Children's Health, Auckland City Hospital, Auckland 1142, New Zealand. [7] Department of Medical Biosciences, Clinical Chemistry, Umeå University, 907 36 Umeå, Sweden.

[8] Current address: Institute of Human Genetics, University Hospital Heidelberg, 69120 Heidelberg, Germany

[9] Department of Paediatric Neurology, University Hospital Essen, 39081 Essen, Germany.

[10]CeGAT GmbH and Praxis für Humangenetik Tübingen, 72076 Tübingen, Germany.

[11] Department of Neurology, Martin-Luther-University Halle-Wittenberg, 06120 Halle (Saale), Germany.

[12] Division for Neurodegenerative Diseases, Department of Neurology, Medical Faculty Mannheim, University of Heidelberg, 68167 Mannheim, Germany.

[13] Department of Child Neurology, Justus Liebig University, 35392 Giessen, Germany.

Correspondence to: Peter Munch Andersen

Department of Clinical Science, Neurosciences, Umeå University, SE-901 85 Umeå, Sweden.

Email: Peter.Andersen@umu.se

Mobile: +46 70 647 6913

Office: +46 90 785 2372

ORCID# 0000-0003-0094-5429

TABLE OF CONTENTS

Supplementary Phenotype Data 4

Supplementary Table 1 – Demographic data of C112X^{Het} individuals 10

Supplementary Table 2 – Clinical presentation of Infantile Superoxide dismutase 1 deficiency syndrome (ISODDES) (extension of Table 1) 11

Supplementary Table 3 – Clinical Chemistry analysis 12

Supplementary Table 4 – Complete blood counts 17

Supplementary Table 5 – Comparison of C112X *SOD1* homozygous ISODDES with D90A *SOD1* homozygous and G127X *SOD1* heterozygous ALS 23

Supplementary Methods 24

Uncropped blots for Figures 3 and 4 26

References 28

I. Supplementary Phenotype Data

Clinical presentations

Family A

The index patient in this family (patient 1) has been described in detail elsewhere (Park *et al.*, 2019). Briefly, the patient developed a progressive loss of motor functions from the 9th month of life onwards. The initially reported symptom was muscular hypotonia. He presented with tetraspasticity, hyperreflexia, inexhaustible myoclonus, and a bilateral Babinski's sign. Intermittent fasciculations, muscular atrophy, and truncal muscular hypotonia were identified as signs of secondary motor neuron involvement. In addition, a positive glabellar tap sign was present while no other signs of frontal release could be noted. The patient was initially started on oral ascorbic acid (up to 200 mg tid) but this was discontinued following nausea and malaise, which quickly resolved after discontinuation. *N*-acetylcysteine (NAC) was then introduced as a redox scavenger and increased stepwise to a final dose of 30 mg/kg BW/d. A clinical reassessment at the age of eight years revealed persistence of the above-mentioned symptoms indicating no change in severity. During periods of emotional stress, the patient showed episodes of cyanosis and perfuse sweating that were self-limiting upon removal of the stressor.

Patient 6, a paternal as well as maternal cousin of patient 1 is the child of consanguineous parents of Afghan origin. The girl was born after an uneventful pregnancy as the fourth child of the union. Muscular hypotonia was noted at the age of six to seven months and was reported to progress over the following months. She was evaluated genetically for spinal muscular atrophy (SMA) with reportedly normal results. After patient 1 was diagnosed, conventional Sanger sequencing of SOD1 identified the c.335dupG (p.C112Wfs*11) mutation in *SOD1*. At the age of five years, she presented with truncal muscular hypotonia in

conjunction with peripheral spasticity. Dysmorphic features were present in the form of lowset ears and overlapping toes.

In this family, no cases of ALS were reported. No symptoms suggestive of neurodegenerative disorders were present at the time of this report in any of the patients' relatives who are heterozygous for the C112X SOD1 mutation.

Family B

The index patient in this family (patient 2) was the subject of a previous description [2]. She is the third child of consanguineous parents of Afghan origin and was born at term via Caesarean section. Although clinically uneventful, the pregnancy was marked by sparse intrauterine movements of the child as per the mother's retrospective assessment. At the age of six months, generalized muscular hypotonia was noted. Over the course of the following year, severe tetraspasticity with brisk deep tendon reflexes and broadened reflex zones developed. Unilateral Babinski's sign (right side) was noted while bulbar signs such as tongue fasciculations and atrophy were absent. Frequent episodes of a pathological autonomous stress response consisting of hyperhidrosis, cyanosis, and periphery vasoconstriction occurred in situations such as blood tapping or emotionally stressful situations. Due to the underlying mutation and hypothesized pathomechanism, oral ascorbate substitution (100 mg tid) was introduced. Following several months of ascorbate substitution, the parents reported improved head control.

At the time of this report, the patient was four years old. She presented with severe spastic tetraparesis with inexhaustible myoclonus and hyperreflexia in combination with axial hypotonia marked by severely reduced albeit slightly improved head control. In comparison to the previous description, an incomplete Babinski sign was noted bilaterally. Furthermore, she developed signs of frontal involvement with affect lability and a Glabellar tap sign. The

parents reported progressive difficulties swallowing liquids, indicating the development of dysphagia.

While no relatives with an ALS-like disorder were reported in this family, the parent's first daughter suffered from a disease with a very similar course to that of the index patient who died at the age of six years. In addition, a second pregnancy was complicated by enhanced nuchal translucency at 13 weeks, oligohydramnion, and hypotrophic kidneys. Chromosomal analysis revealed a normal female karyotype. However, this pregnancy was terminated at 21 weeks.

Family C

Patient 3 is the fourth child of consanguineous parents of Afghan origin. The girl was born spontaneously at term after an uneventful pregnancy. The parents reported appropriate development – also when compared to the four older siblings – for the first months of life. Standardized pediatric examinations during the first months of life identified no abnormalities. Directed gripping was possible at the age of 6-7 months, crossing the midline approximately 1 month later. The patient was able to sit without support at the age of 5-6 months.

At the age of 10 months, the patient suffered a febrile respiratory infection. Following this, generalized muscular hypotonia developed and the patient regressed in the previously mentioned milestones of motor development. At the time of evaluation, the patient was four years old. She presented with severe truncal muscular hypotonia with no head control, which was accompanied by severe spasticity of the extremities with hyperreflexia and vastly broadened reflex zones. Bilateral Babinski's sign was present while other pyramidal tract signs were not noted. In addition to these signs of upper motor neuron involvement, the girl showed lower motor neuron signs consisting of generalized muscular atrophy and intermittent

fasciculations. Severe dysphagia as well as tongue atrophy and fasciculations were bulbar symptoms seen in this patient. No signs of frontal involvement were noted. Ophthalmological examination revealed bilateral optic nerve atrophy.

Family D

Patients 4 and 5 are the children of a consanguineous couple (first degree cousins with multiple consanguineous unions on both sides of the family) of Afghan origin. Patient 4 is a 13-year-old girl who was born after an uneventful pregnancy. The initial development was reported to have been regular. According to the parents, motor impairment was first noted around the age of one year, when the patient was able to walk with support but began showing signs of muscular hypotonia. Her condition deteriorated over the course of several months and the patient developed tetraspasticity with truncal hypotonia resulting in poor head control.

At the age of 13 years, the patient presented with pronounced tetraspasticity in conjunction with truncal muscular hypotonia. Severe spastic dysarthria led to impairment of verbal communication. In term of UMNS, unilateral Babinski's sign, pronounced hyperreflexia, and bilateral loss of the abdominal reflex were present. Signs of LMNS consisted of generalized muscular atrophy, muscular hypotonia, and intermittent fasciculations. Furthermore, dysphagia as well as tongue atrophy and fasciculations indicated bulbar involvement. Following several years of disease course, the patient began to develop intermittent episodes of inappropriate laughter and other emotional reactions. However, no frontal release signs were seen in the physical examination. Despite initially age-adequate development of urinary continence, incontinence developed at the age of 13 years. Fecal continence was preserved although the patient has been suffering from chronic obstipation since early childhood.

Patient 5, the younger brother of patient 4, was born at a gestational age of 30 weeks followed by complicated postnatal adaptation, which reportedly resolved without sequelae.

Delayed motor development was noted at the age of approximately 12 months. At the age of 10 years, he presented with symmetric bilateral spasticity and hyperreflexia. Truncal hypotonia and generalized muscular atrophy in conjunction with intermittent fasciculations were also noted. Bulbar symptoms included dysphagia and spastic dysarthria, making verbal communication impossible. Similarly, to the symptoms observed in his sister, the boy developed inappropriate emotional reactions at the age of 9-10 years. The glabellar reflex, a frontal release sign, was an additional indication of frontal lobe involvement. Urinary and fecal incontinence were also seen, as was an inappropriate stress response consisting of hyperhydrosis and facial discoloration.

No diagnosed cases of ALS were reported in this family. A maternal great-grandfather of the patients was reported to have suffered from a sudden onset of paresis in one shoulder, spreading distally and eventually rendering him bedridden. He reportedly died shortly afterwards. No diagnosis was made at the time and no medical records were available for review.

Family E

Patients 7 and 8 are the children of consanguineous parents of Afghan origin.

Patient 7 is a currently 9-year-old girl who was born at term after an uneventful pregnancy with reportedly normal development until the age of 5 months, when she began to exhibit muscle weakness. No sitting or head control were achieved, and the patient remains averbal. At the time of evaluation, she exhibited severe truncal hypotonia in conjunction with limb spasticity and multiple contractures requiring the use of a wheelchair. Bulbar symptoms in the form of dysphagia were present. Of note, optic atrophy was noted in ophthalmological examination.

Patient 8 was equally born after an uneventful pregnancy followed by a normal postnatal adaptation. Symptoms were first noted within the first year of life, with pronounced head lag and truncal muscular hypotonia in addition to limb hypertonia. At the time of evaluation, bilateral lower limb spasticity accompanied by truncal hypotonia was detected. No verbal expression except babbling was possible. RMI imaging at the age of 2 years identified hydrocephalus requiring ventriculo-peritoneal (VP) shunt treatment. Both patients exhibited distress-provoked breath-holding type spells with extensor posturing akin to decerebrate posture that resolved spontaneously. Initially interpreted in patient 8 as seizures, these were treated with levetiracetam to no avail and treatment was discontinued.

II. Supplementary Table 1.
Demographic data of C112X^{Het} individuals

	**parents**							**siblings**				
**Individual**	1.2	1.3	2.2	2.3	3.2	3.3	4.2	4.3	4.4	3.4	3.5	3.6
**Family**	A	A	B	B	C	C	D	D	A	C	C	C
**Sex**	male	female	male	female	male	female	male	female	male	female	female	female
**Age**	45	36	41	35	40	35	38	31	14	15	14	12
**Relation to index in family**	father	mother	father	mother	father	mother	father	mother	brother	sister	sister	sister

III. Supplementary Table 2.

Clinical presentation of Infantile Superoxide dismutase 1 deficiency syndrome (Extension of Table 1)

	Patient 1[1]	Patient 2[2]	Patient 3	Patient 4	Patient 5	Patient 6	Patient 7	Patient 8
**Family**	A	B	C	D	D	A	E	E
**Sex**	male	female	female	female	male	female	female	male
**Age at evaluation**	8 years	4 years	4 years	13 years	10 years	5 years	9 years	3 years
**Reported age of onset**	9 months	6 months	10 months	9 - 12 months	approx. 12 months	6-7 months	5 months	5 months
**Initial symptom**	truncal muscular hypotonia<br>delayed motor development	generalized muscular hypotonia	generalized muscular hypotonia following febrile respiratory infection	truncal muscular hypotonia<br>delayed motor development	lack of motor development	lack of motor development	muscular hypotonia, developmental delay	lower limb spasticity
**Current GMFCS - E&R level**	V	V	V	V	V	V	V	V
**Communication**	spastic dysarthria<br>non-verbal communication<br>pointing, head movements	spastic dysarthria<br>vocalizations<br>non-verbal communication<br>pointing, head movements	spastic dysarthria	spastic dysarthria<br>single words<br>non-verbal communication<br>pointing, head movements, use of assisting device (tablet computer)	severe spastic dysarthria<br>non-verbal communication<br>pointing, head movements		never developed sords, babbling then averbal<br>non-verbal communication<br>eye contact, head movements	babbled then averbal<br>non-verbal communication<br>eye contact, vocalisation, pointing
**Milestones of motor development**	head control:<br>rolling over:<br>sitting:<br>crawling: 9 months<br>walking with support: -<br>walking unsupported: -	head control: 3 months<br>rolling over: 6 months<br>sitting: -<br>crawling: -<br>walking with support: -<br>walking unsupported: -	head control: -<br>rolling over: -<br>gripping: 6-7 months, lost at the age of 10 months<br>crossing the midline: 7-8 months, lost at the age of 10 months<br>sitting: 5-6 months, lost at the age of 10 months<br>walking with support: -<br>walking unsupported: -	head control: -<br>rolling over: -<br>sitting: 7 months<br>crawling: N/A<br>walking with support: 12 months<br>walking unsupported: -	head control: -<br>rolling over: -<br>sitting: 6 months<br>crawling: N/A<br>walking with support: -<br>walking unsupported: -	head control: -<br>rolling over: N/A<br>sitting: N/A<br>walking with support: -<br>walking unsupported: -	head control: -<br>never rolled over or sat or crawled, never walked<br>sitting: -<br>crawling: -<br>walking with support: -<br>walking unsupported: -	head control: -<br>briefly able to sit, stand, supported walking (around 1yo) then all regressed, now no head control, cannot sit supported or crawl<br>sitting: -<br>crawling: -
**Upper motor neuron symptoms**	Babinski's sign L +, R +<br>Gordon's sign L -, R -<br>Oppenheim's sign L -, R -<br>bilateral spasticity<br>inexhaustible myoclonus<br>hyperreflexia<br>hyperreactive tendon reflexes<br>broadened reflex zones<br>bilateral loss of the abdominal reflex	Babinski's sign L +, R +<br>Gordon's sign L -, R -<br>Oppenheim's sign L -, R -<br>spastic tetraparesis<br>inexhaustible myoclonus<br>hyperreflexia<br>hyperreactive tendon reflexes<br>broadened reflex zones	Babinski's sign L +, R +<br>Gordon's sign L -, R -<br>Oppenheim's sign L -, R -<br>bilateral spasticity<br>hyperreflexia<br>brisk, hyperreactive tendon reflexes<br>broadened reflex zones	Babinski's sign L +, R -<br>Gordon's sign L - , R -<br>Oppenheim's sign L -, R -<br>bilateral spasticity<br>hyperreflexia<br>hyperreactive tendon reflexes<br>broadened reflex zones<br>bilateral loss of the abdominal reflex	Babinski's sign L +, R +<br>Gordon's sign L -, R -<br>Oppenheim's sign L +, R -<br>bilateral spasticity<br>hyperreflexia<br>hyperreactive tendon reflexes<br>broadened reflex zones<br>bilateral loss of the abdominal reflex	bilateral spasticity	spastic quadriplegia	spasttic quadripleagia
**Lower motor neuron symptoms**	truncal muscular hypotonia<br>mild muscular atrophy	axial hypotonia	severe muscular hypotonia<br>generalized muscular atrophy<br>intermittent fasciculations	muscular hypotonia<br>lack of head control<br>generalized muscular atrophy<br>intermittend fasciculations	severe muscular hypotonia<br>truncal hypotonia<br>generalized muscular atrophy<br>intermittent fasciculations	muscular hypotonia<br>truncal hypotonia	severe muscular hypotonia<br>truncal hypotonia<br>lack of head control	severe muscular hyptonia<br>truncal hypotonia<br>lack of head control
**Bulbar symptoms**	dysphagia necessitating gastrostomy tube feeding	dysphagia<br>siallorrhea	severe dysphagia necessitating gastrostomy tube feeding<br>spastic dysarthria<br>sialorrhea<br>tongue atrophy<br>tongue fasciculations	mild dysphagia<br>spastic dysarthria<br>tongue atrophy<br>tongue fasciculations	dysphagia<br>spastic dysarthria<br>sialorrhea<br>tongue atrophy<br>tongue fasciculations	N/A	multiphase dysphagia	multiphase dysphagia
**Frontal lobe symptoms**	Glabellar tap sign	Glabellar tap sign<br>intermittent inadequate fits of crying	-	intermittent inadequate fits of laughter	Glabellar tap sign<br>intermittent inadequate fits of laughter, onset at 9 years of age	N/A	-	-
**Excessive startle reaction**	+	-	-	-	-		provoked intermittent self-resolving tonic decerebrate posture akin to breath-holding spells, triggered by distress	provoked intermittent self-resolving tonic decerebrate posture akin to breath-holding spells, triggered by distress
**Autonomic symptoms**	urinary and fecal incontinence<br>pathological autonomous stress response<br>intermittent hyperhidrosis	urinary and fecal incontinence<br>pathological autonomous stress response<br>hyperhidrosis<br>cyanosis<br>peripheral vasoconstriction	not applicable<br>(formal diagnosis of incontinence not possible due to young age)	urinary incontinence<br>age appropriate continence developed, lost at 13y of age<br>chronic obstipation	urinary and fecal incontinence<br>pathological autonomous stress response<br>hyperhidrosis<br>facial dyscoloration	N/A		-
**Ocular symptoms**	-	-	bilateral optic nerve atrophy	-	-	N/A	bilateral optic atrophy	-
**Skeletal symptoms**	bilateral hip luxation<br>neuromuscular scoliosi	bilateral hip luxation	thoracolumbar scoliosis<br>pes equinus	severe thoracolumbar scoliosis<br>bilateral hip luxation<br>osteopenia<br>multiple pathological fractures<br>ulnar deviation	thoracolumbar scoliosis<br>hip luxation (right)<br>osteopenia<br>congenital radial head dislocation (right)<br>recurring pathological fractures<br>ulnar deviation	bilateral hip luxation	thoracolumbar kypho-scoliosis<br>bilateral hip subluxation<br>multiple femoral head fractures	-
**Dysmorphic features**	low set, posteriorly rotated ears<br>overlapping toes	broad nasal bridge		low set ears<br>overlapping toes	low set ears<br>overlapping toes<br>arched palate	low set ears<br>overlapping toes		simplified outer helix of the right ear<br>hydrocephalus
**Miscellaneous symptoms**								

[1] Park et al. 2019, [2] Andersen et al. 2019
"+": positive / present; "-": negative / not achieved; "N/A": not available

IV. Supplementary Table 3 – Clinical Chemistry analysis

		C112X[Hom]	C112X[Het]	*P*-value[1]
		Statistics presented: n/N (%); Mean (SD)		
	**N**	5	12	
	**Sex**			
	Female	3 / 5 (60 %)	7 / 12 (58 %)	
	Male	2 / 5 (40 %)	5 / 12 (42 %)	
**Liver**	**P-ASAT**<br>µkat/L<br><br>*Reference:*<br>*1-5 y: < 0.93*<br>*5-9 y: < 0.8*<br>*9-18y: < 0.72*<br>*Adult F: < 0.6*<br>*Adult M: < 0.75*	0.55 (0.17)	0.46 (0.12)	0.23
	**P-ALAT**<br>µkat/L<br><br>*Reference:*<br>*F 6m – 9y: < 0.39*<br>*M 6m – 9y: < 0.29*<br>*9 – 18y: < 0.51*<br>*Adult F: < 0.75*<br>*Adult M: < 1.1*	0.62 (0.48)	0.46 (0.37)	0.57
	**P-GGT**<br>µkat/L<br><br>*Reference:*<br>*6 m – 8 y: < 0.3*<br>*8 – 13 y: < 0.4*<br>*13 – 18 y: < 0.6*<br>*Adult Female: < 0.75*<br>*Adult Male: < 1.3*	0.22 (0.06)	0.4 (0.28)	0.06
	**P-ALP**<br>µkat/L<br><br>*Reference:*<br>*2 – 8 y: 2.0 – 5.0*<br>*9 – 14 y: 1.4 – 8.7*<br>*F 15 – 17 y: 0.7 – 4.0*<br>*M 15 – 17 y: 1.2 – 5.6*<br>*Adult: 0.7 – 1.9*	2.45 (0.68)	2.16 (1.83)	0.33
	**P-Total bilirubin**<br>µmol/l<br><br>*Reference:*<br>*6 m – 7 y: < 8*<br>*8 – 12 y: < 14*<br>*13 – 18 y: < 30*<br>*Adult: < 25*	17.6 (12.8) [2]	8.4 (4.0)	0.06
	**P-Conjugated bilirubin**<br>µmol/L<br><br>*Reference:*<br>*0 m – 8 y: < 4*<br>*8 – 13 y: <6*<br>*13 – 18 y: < 12*<br>*Adult: < 5*	3.08 (1.91)	1.49 (0.98)	0.07
	**P-Ammonia**<br>µmol/L<br><br>*Reference:*<br>*F: < 51*<br>*M: < 60*	35 (22.86)	26.92 (7.92)	>0.9
	**P-Bile acids**<br>µmol/L<br><br>*Reference:*<br>*< 10*	4.08 (4.13)	4.4 (2.06)	0.44
	**P-Albumin**<br>g/L<br><br>*Reference:*<br>*4 d – 12 y: 38-47*<br>*12 – 18 y: 41-50*<br>*Adult: 36 – 48*	43.2 (3.7)	43.9 (5.0)	0.52

**Lipids**	**P-Triglycerides** mmol/L<br><br>*Reference:*<br>*6 m – 18 y: < 2.9*<br>*Adult: < 2.6*	1.07 (0.7)	1.86 (1.3)	0.1
	**P-Cholesterol** mmol/L<br><br>*Reference:*<br>*F 6 m – 3 y: 1.9 – 5.1*<br>*M 6 m – 3 y: 2.8 – 5.7*<br>*F 3 – 18 y: 3.2 – 6.1*<br>*M 3 – 18 y: 2.9 – 6.0*<br>*19 – 30 y: 2.9 – 6.1*<br>*31 – 50 y: 3.3 – 6.9*	4.05 (0.44)	4.57 (1.23)	0.65
	**P-APO A** g/L<br><br>*Reference:*<br>*F: 1.1 – 2.3*<br>*M: 1.0 – 2.1*	1.57 (0.34)	1.54 (0.29)	0.88
	**P-APO B** g/L<br><br>*Reference:*<br>*0.66 – 1.67*	0.72 (0.12)	0.91 (0.33)	0.38
**Kidney**	**P-Cystatin C** mg/L<br><br>*Reference:*<br>*1 – 49 y: 0.6 – 1.1*	0.78 (0.15)	0.84 (0.11)	0.27
	**P-Creatinine** µmol/L<br><br>*Reference:*<br>*6 m – 2 y: 18 – 48*<br>*2 – 6 y: 22 – 53*<br>*F 6 – 11 y: 31- 70*<br>*M 6 – 11 y: 31 – 76*<br>*F 11 – 15 y: 36-70*<br>*M 11 – 15 y: 44 – 90*<br>*F 15 – 18 y: 49 – 86*<br>*M 15 – 18 y: 55 – 106*<br>*Adult F: 45 – 90*<br>*Adult M: 60 – 105*	25 (6)	56 (15)	**<0.001**
	**P-Urea** mmol/L<br><br>*Reference:*<br>*1 – 10 y: 3.1 – 7.8*<br>*11 – 18 y: 2.7 – 7.1*<br>*Adult F: 2.6 – 6.4*<br>*Adult M: 3.2 – 8.1*	4.06 (1.53)	4.75 (1.3)	0.44
	**P-Urate** µmol/L<br><br>*Reference:*<br>*6 m – 9y: 160 – 350*<br>*9 – 13 y: 130 – 350*<br>*F 13 – 18 y: 110 – 340*<br>*M 13 – 18 y: 170 – 470*<br>*Adult F: 155 – 350*<br>*Adult M: 230 – 480*	152 (38)	237 (84)	**0.05**
	**U-Albumin/creatinine ratio**<br><br>*Reference:*<br>*1 – 5 y: < 3.3*<br>*6 – 10 y: < 2.7*<br>*11 – 15 y: < 2.1*<br>*> 16 y: < 3.0*	1.65 (1.52)	1.00 (1.37)	0.23
	**U-α1-microglobulin/creatinine ratio**<br><br>*Reference:*<br>*< 0.7 g/mol*	Low or below the limit of detection, indicating normal protein reabsorption in the prox. tubules		-

Electrolytes	**P-Na⁺** mmol/L<br><br>*Reference: 137-145*	138.38 (1.2)	137.88 (1.67)	0.56
	**P-K⁺** mmol/L<br><br>*Reference:*<br>*1 – 18 y: 3.7 – 4.8*<br>*> 18 y: 3.5 – 4.4*	4.22 (0.54)	4.38 (0.42)	0.57
	**P-Cl⁻** mmol/L<br><br>*Reference:*<br>*0 – 18 y: 103 – 111*<br>*Adult: 100 – 110*	103.9 (2.47)	10.273 (1.86)	0.4
	**P-Mg²⁺** mmol/L<br><br>*Reference:*<br>*0 – 18 y: 0.76 – 1.0*<br>*Adult: 0.7 – 0.95*	0.87 (0.07)	0.86 (0.05)	0.72
	**P-Ca²⁺** mmol/L<br><br>*Reference:*<br>*1 – 18 y: 2.32 – 2.67*<br>*Adult: 2.15 – 2.5*	2.46 (0.17)	2.4 (0.08)	0.56
	**P-PO₄** mmol/L<br><br>*Reference:*<br>*1 – 7 y: 1.35 – 2.03*<br>*F 8 – 12 y: 1.11 – 1.83*<br>*M 8 – 12 y: 1.28 – 1.99*<br>*F 13 – 18 y: 0.94 – 1.63*<br>*M 13 – 18 y: 0.94 – 1.8*<br>*Adult F: 0.8 – 1.5*<br>*Adult M: 0.7 – 1.6*	1.7 (0.23)	1.2 (0.08)	**0.004**
Adrenal gland	**P-ACTH** pmol/L<br><br>*Reference: 01.05.14*	4.4 (7.0)	5.4 (5.5)	**0.05**
	**P-Cortisol** nmol/L<br><br>*Reference: 135 – 540*	251 (162)	271 (149)	0.88
Heart	**P-Troponin T (hs)** ng/L<br><br>*Reference: < 15*	5.33 (0.53)	4.69 (1.9)	0.13
	**P-NTproBNP** ng/L<br><br>*Reference:*<br>*F: < 150*<br>*M: < 100*	56 (41)	50 (61)	0.38
Skeletal muscle	**P-CK** μkat/L<br><br>*Reference:*<br>*0 – 2 y: < 3.8*<br>*2 – 10 y: < 3.8*<br>*10 – 14 y: < 6.6*<br>*14 – 18 y: < 9.0*<br>*Adult M: < 6.7*<br>*Adult F: < 3.5*	1.55 (1.15)	2.33 (1.28)	0.28

Thyroid gland	**P-TSH**<br>mIU/L<br><br>*Reference:*<br>*0.27 – 4.2*	1.68 (1.04)	2.56 (1.09)	0.23
	**P-fT3**<br>pmol/L<br><br>*Reference:*<br>*< 6.8*	5.78 (1.08)	5.14 (1.23)	0.23
	**P-fT4**<br>pmol/L<br><br>*Reference:*<br>*12 – 22*	18.84 (1.19)	14.07 (2.08)	**<0.001**
**Parathyroid gland & bone metab**	**P-PTH**<br>pmol/L<br><br>*Reference:*<br>*1.6 – 6.9*	3.4 (1.6)	7.7 (7.0)	0.06
**Pancreas**	**P-Amylase**<br>µkat/L<br><br>Reference:<br>2 -15 y: 0.1 – 0.6<br>15 – 18 y: 0.16 – 0.8<br>Adult: 0.15 – 1.1	0.27 (0.11)	0.38 (0.17)	0.23
	**P-Insulin**<br>mIU/L<br><br>*Reference:*<br>*2.6 – 24.9*	6 (2)	17 (15)	**0.001**
	**P-C-peptide**<br>nmol/L<br><br>Reference:<br>0.37 – 1.47	0.49 (0.05)	1.06 (0.44)	**<0.001**
	**P-Fructosamine**<br>µmol/L<br><br>*Reference:*<br>*160-340*	301.5 (56.2)	288.8 (38.1)	**0.91**
	**B-HbA1c** mmol/mol<br><br>*Reference:*<br>*< 50 y: 27 – 42*<br>*> 50 y: 31 – 46*	16.4 (2.4)	33.2 (3.4)	**<0.001**
**Iron metabolism**	**P-Ferritin**<br>µg/L<br><br>*Reference:*<br>*5 m – 14 y: 15 – 200*<br>*F 15 – 50 y: 15 – 150*<br>*M > 14 y: 30 – 400*<br>*F > 50 y: 30 - 400*	165 (290)	104 (112)	>0.9
	**P-Iron**<br>µmol/L<br><br>*Reference:*<br>*1 – 14 y: 9 – 22*<br>*> 14 y: 9 – 34*	11.2 (3.9)	13.1 (4.3)	0.56
	**P-Transferrrin**<br>g/L<br><br>*Reference:*<br>*6 m – 12 y: 2.1 – 3.5*<br>*F 12 – 18 y: 2.4 – 4.3*<br>*M 12 – 18 y: 2.4 – 3.8*<br>*Adult: 1.87 – 3.19*	2.67 (0.46)	3.09 (0.54)	0.23
**Vitamins**	**P-Folate**<br>nmol/L<br><br>*Reference:*<br>*7 – 39*	21 (16)	22 (10)	0.52
	**P-Vitamin B_{12}**<br>pmol/L<br><br>*Reference:*<br>*145 – 569*	475 (170)	264 (67)	**0.006**

Immune system & inflammation	**P-IgG**<br>g/L<br><br>*Reference:*<br>*2 – 3 y: 3.7 – 11.8*<br>*3 – 4 y: 4.5 – 12.8*<br>*4 – 5 y: 5.1 – 13.7*<br>*5 – 6 y: 5.6 – 14.3*<br>*6 – 7 y: 6.0 – 14.7*<br>*7 – 8 y: 6.3 – 15.0*<br>*8 – 9 y: 6.5 – 15.1*<br>*9 – 10 y: 6.6 – 15.2*<br>*10 – 11 y: 6.6 – 15.3*<br>*11 – 12 y: 6.7 – 15.3*<br>*12 – 13 y: 6.7 – 15.5*<br>*13 – 14 y: 6.8 – 15.7*<br>*> 14 y: 6.7 – 14.5*	9.79 (1.52)	12.42 (2.26)	**0.02**
	**P-IgA**<br>g/L<br><br>*Reference:*<br>*2 – 3 y: 0.1 – 1.3*<br>*3 – 4 y: 0.2 – 1.6*<br>*4 – 5 y: 0.3 – 1.9*<br>*5 – 6 y: 0.3 – 2.1*<br>*6 – 7 y: 0.4 – 2.2*<br>*7 – 8 y: 0.4 – 2.4*<br>*8 – 10 y: 0.4 – 2.5*<br>*10 – 11 y: 0.3 – 2.6*<br>*11 – 13 y: 0.3 – 2.7*<br>*13 – 14 y: 0.2 – 2.8*<br>*> 14 y: 0.9 – 4.5*	1.51 (0.61)	1.69 (0.82)	0.88
	**P-IgM**<br>g/L<br><br>*Reference:*<br>*F 2 – 3 y: 0.3 – 2.1*<br>*M 2 – 3 y: 0.3 – 1.8*<br>*F 3 – 5 y: 0.4 – 2.2*<br>*M 3 – 5 y: 0.3 – 1.9*<br>*F 6 – 7 y: 0.5 – 2.4*<br>*M 6 – 7 y: 0.4 – 2.0*<br>*F 7 – 9 y: 0.5 – 2.5*<br>*M 7 – 9 y: 0.4 – 2.0*<br>*F 9 – 10 y: 0.6 – 2.6*<br>*M 9 – 12 y: 0.4 – 2.1*<br>*F 10 – 11 y: 0.6 – 2.7*<br>*F 11 – 12 y: 0.6 – 2.8*<br>*F 12 – 13 y: 0.7 – 2.9*<br>*M 12 – 14 y: 0.5 – 2.2*<br>*F 13 – 14 y: 0.7 – 3.0*<br>*> 14 y: 0.3 – 2.1*	0.91 (0.26)	0.96 (0.5)	>0.9
	**P-CRP**<br>mg/L<br><br>*Reference:*<br>*< 3*	0.6 (0.33)	1.04 (0.87)	0.38
	**P-Haptoglobin**<br>g/L<br><br>*Reference:*<br>*0 – 6 y: 0.1 – 1.0*<br>*F 6 – 10: 0.15 – 1.2*<br>*M 6 – 10: 0.1 – 1.0*<br>*F 10 – 12: 0.1 – 1.35*<br>*M 10 – 14: 0.1 – 1.2*<br>*F 12 – 14: 0.1 – 1.1*<br>*F 14 – 16: 0.15 – 1.25*<br>*M 14 – 16: 0.2 – 1.5*<br>*Adult: 0.24 – 1.9*	0.56 (0.28)	0.87 (0.36)	0.13
	[1] Comparison of C112X^{Hom} with C112X^{Het} using Mann-Whitney U test<br><br>[2] Patient 4 showed elevated bilirubin levels without any additional signs of liver damage and consistent with variants of bilirubin metabolism such as Gilbert's syndrome. The mean bilirubin level of C112X^{Hom} individuals excluding patient 4 is 12.0 ± 1.7 µmol/L			
	P – plasma, U – urine, B – whole blood<br>F – female, M – male, m – months, y – year(s)			

V. Supplementary Table 4. Complete blood counts

Patient 1

Age	Erc count (x 10^6/µl)	MCV fl	Hkt %	Hb (g/L)	Reti (rel., %)	Reti (abs., x 10^3/µl)	Reti He (pg)	RPI	Neutrophils (x 10^3/µl)	Lymphocytes (x 10^3/µl)	Monocytes (x 10^3/µl)	Eosinophils (x 10^3/µl)	Basophils (x 10^3/µl)	Mean Platelet volume (fL)	Immature granulocytes (x 10^3/µl)
8y2m	3.39<br>(3.96-5.03)	97.1<br>(74.4-86.1)	32.9<br>(32.2-39.8)	111<br>(107-134)	1.8<br>(1-1.9)	60<br>(42-70)	37 pg<br>(23.6-33.9)	0.9<br>2-3	5.31<br>(4.31-11)	217<br>(206-369}	3.94<br>(1.63-7.75)	0.99<br>(0.97-3.96)	0.01<br>(0.01-0.06)	13.0<br>(9.2-11.4)	0.03<br>(<0.04)
7y3m	3.68<br>(3.96-5.03)	86.7<br>(74.4-86.1)	31.9<br>(32.2-39.8)	108<br>(107-134)	NA	NA	NA	NA	5.93<br>(4.31-11)	187<br>(206-369)	3.2<br>(1.63-7.75)	1.99<br>(0.97-3.96)	0.02<br>(0.01-0.06)	12.2<br>(9.2-11.4)	0.01<br>(<0.04)
7y0m	3.72<br>(3.96-5.03)	90.3<br>(74.4-86.1)	33.6<br>(32.2-39.8)	109<br>(107-134)	NA	NA	NA	NA	7.30<br>(4.31-11)	219<br>(206-369)	NA	NA	NA	NA	NA
6y10m	3.71<br>(3.96-5.03)	88.9<br>(74.4-86.1)	33.0<br>(32.2-39.8)	107<br>(107-134)	NA	NA	NA	NA	5.86<br>(4.31-11)	227<br>(206-369)	2.93<br>(1.63-7.75)	2.18<br>(0.97-3.96)	0.01<br>(0.01-0.06)	12.3<br>(9.2-11.4)	0.01<br>(<0.04)
6y9m	3.62<br>(3.96-5.03)	89.5<br>(74.4-86.1)	32.4<br>(32.2-39.8)	107<br>(107-134)	1<br>(1-1.9)	37.3<br>(42-70)	33.1<br>(23.6-33.9)	0.5	6.23<br>(4.31-11)	213<br>(206-369)	3.2<br>(1.63-7.75)	2.3<br>(0.97-3.96)	0.01<br>(0.01-0.06)	12.2<br>(9.2-11.4)	0.01<br>(<0.04)
6y1m	3.59<br>(3.96-5.03)	91.4<br>(74.4-86.1)	32.8<br>(32.2-39.8)	108<br>(107-134)	NA	NA	NA	NA	5.30<br>(4.31-11)	218<br>(206-369)	NA	NA	NA	NA	NA
5y11m17d	3.56<br>(3.89-4.97)	90.4<br>(71.3-84)	32.2<br>(31-37.7)	106<br>(102-127)	NA	NA	NA	NA	4.40<br>(5.14-13.8)	197<br>(202-403)	NA	NA	NA	NA	NA
5y11m11d	3.67<br>(3.89-4.97)	85.5<br>(71.3-84)	31.5<br>(31-37.7)	107<br>(102-127)	NA	NA	NA	NA	5.06<br>(5.14-13.8)	198<br>(202-403)	3.27<br>(1.54-7.92)	1.39<br>(1.13-5.2)	0.02<br>(0.01-0.06)	12.1<br>(9-10.9)	0.01<br>(<0.06)
3y10m	3.86<br>(3.89-4.97)	88.6<br>(71.3-84)	34.2<br>(31-37.7)	112<br>(102-127)	NA	NA	NA	NA	7.34<br>(5.14-13.8)	322<br>(202-403)	2.4<br>(1.54-7.92)	3.81<br>(1.13-5.2)	0.03<br>(0.01-0.06)	11.7<br>(9-10.9)	0.02<br>(<0.06)

Patient 2

Age	Erc count (x 10^6/µl)	MCV fL	Hkt %	Hb g/L	Reti (rel., %)	Reti (abs., x 10^9/L	RPI	Leukocytes (x 10^9/L)	Thrombocytes (x 10^9/L)	Neutrophils (x 10^9/L)	Lymphocytes (x 10^9/L)	Monocytes (x 10^9/L)	Eosinophils (x 10^9/L)	Basophiles (x 10^9/L)
4y0m	4.05	83	33.5	107	1.7	67	NA	6.1	327	2.64	2.65	0.34	0.31	<0.1
2y7m	4.44	79	34.9	113	NA	NA	NA	10.2	398	3.81	4.51	0.72	0.49	0.12
2y5m	4.4	79	34.8	109	3.2	141	1.7	9.5	447	3.35	5.23	0.54	0.36	**<0.1**
2y4m	4.28	77	33	11	NA	NA	NA	13.6	436	5.31	6.46	0.76	0.4	0.1
1y11m	4.11	75	30.8	98	NA	NA	NA	4.7	368	1.98	2.05	0.28	0.23	<0.1
1y4m15d	4.08	81.7	33.3	100	NA	NA	NA	9.8	689	NA	NA	NA	NA	NA
1y4m2d	3.6	80.7	29.1	106	1.4	51	0.5	7.5	249	3.42	3.07	0.62	<0.1	<0.1
0y9m	4.09	86.8	35.5	111	NA	NA	NA	13.9	496	3.71	8.28	0.98	0.37	<0.1
**reference interval**	3.1-5.3	70.0-86.0	30-43	101-131	0.5-2.0	25-100	<2	(6.0-17.5	150-500	1.5-8.7	3.5-14.5	0-1.2	0-0.7	0-0.1

Patient 3

Age	Erc count (x 10^{12}/L)	MCV fL	Hkt %	Hb g/L	Reti (rel., %)	Reti (abs., x 10^3/µl)	RPI	Leukocytes (x 10^9/L)	Thrombocytes (x 10^9/L)	Neutrophils (x 10^9/L)	Lymphocytes (x 10^9/L)	Monocytes (x 10^9/L)	Eosinophils (x 10^9/L)	Basophiles (x 10^9/L)
4y6m	3.5	97	34	103	NA	NA	NA	6.4	312	4.09	1.81	0.4	0.11	0.01
**reference interval**	3.85-5.15	73-91	32.5-41.5	107-139				5.4-13.8	200-460	1.5-8.5	2.2-8.5	0.1-1.1	0.02-0.75	0-0.2

Patient 4

Age	Erc count (x 10^6/µL)	MCV (fL)	Hkt (%)	Hb (g/L)	Reti (rel., %)	Reti (abs., x 10^3/µL)	Reti He (pg)	RPI	Leukocytes (x 10^3/µL)	Thrombocytes (x 10^3/µL)	Neutrophils (x 10^3/µL)	Lymphocytes (x 10^3/µL)	Monocytes (x 10^3/µL)	Eosinophils (x 10^3/µL)	Basophiles (x 10^3/µL)	Mean Platelet volume (fL)	Immature granulocytes (x 10^3/µL)
13y9m	2.66	93.2	24.8	88	3.1	82.7	35.5	0.9	4.69	136	2.79	1.54	0.22	0.09	0.05	12.6	0.01
reference interval	3.93-4.9	76.9-90.6	33.4-40.4	108-133	0.9-1.5	42-65	28.2-33.9	44622	4.19-9.43	194-345	1.82-7.47	1.16-3.33	0.19-0.72	0.02-0.32	0.01-0.05	9.6-11.7	<0.03

Patient 5

Age	Erc count (x 10^6/µL)	MCV fL	Hkt (%)	Hb (g/L)	Reti (rel., %)	Reti (abs., x 10^3Tsd/µL)	Reti He (pg)	RPI
10y9m	NA	NA	32.9	107	NA	NA	NA	NA
reference range			(32.2-39.8)	(107-134)				

Patient 6

Age	Erc count (x 10^6/µL)	MCV fL	Hkt (%)	Hb (g/L)	Reti (rel., %)	Reti (abs., x 10^3Tsd/µL)	Reti He (pg)	RPI
no hematology data available								

Patient 7

Age	Erc count (x 10^6/µL)	MCV (fL)	Hkt (%)	Hb (g/L)	Reti (rel., %)	Reti (abs., x 10^3/µL)	Reti He (pg)	RPI	Leukocytes (x 10^3/µL)	Thrombocytes (x 10^3/µL)	Neutrophils (x 10^3/µL)	Lymphocytes (x 10^3/µL)	Monocytes (x 10^3/µL)	Eosinophils (x 10^3/µL)	Basophiles (x 10^3/µL)	Mean Platelet volume (fL)	Immature granulocytes (x 10^3/µL)
9y11m	3.73	95	36	109	NA	NA	NA	NA	5.01	223	2.4	2.05	0.39	0.14	0.03	12.6	0.01
9y6m	3.82	97	37	115	NA	NA	NA	NA	11.66	235	10.85	0.38	0.4	0.15	0.03	11.7	0.05
9y5m	3.32	94	31	100	NA	NA	NA	NA	6.13	208	3.98	1.51	0.45	0.17	0.04	11.8	0.03
9y4m	3.79	95	36	114	NA	NA	NA	NA	4.48	221	2.08	1.84	0.35	0.15	0.04	12.4	0.01
8y11m	3.42	94	32	104	NA	NA	NA	NA	5.52	341	2.81	2.31	0.25			11.8	0
reference range	4.2-5.6	75-90	35-43	115-145					4.3-12.0	150-425	1.5-7.0	1.4-4.5	0.3-0.9	0.0-0.9	0.0-0.1	8.5-12.0	0.00-0.06

Patient 8

Age	Erc count (x 10^6/µL)	MCV (fL)	Hkt (%)	Hb (g/L)	Reti (rel., %)	Reti (abs., x 10^3/µL)	Reti He (pg)	RPI	Leukocytes (x 10^3/µL)	Thrombocytes (x 10^3/µL)	Neutrophils (x 10^3/µL)	Lymphocytes (x 10^3/µL)	Monocytes (x 10^3/µL)	Eosinophils (x 10^3/µL)	Basophiles (x 10^3/µL)	Mean Platelet volume (fL)	Immature granulocytes (x 10^3/µL)
2y9m	3.64	85	31	92	NA	NA	NA	NA	4.3	311	1.6	2	0.3	0.2	0	9.7	0.01
2y5m28d	2.96	82	24	76	NA	NA	NA	NA	6.07	327	4.31	1.26	0.35	0.12	0.03	10.4	0.01
2y5m26d	3.36	82	28	84	NA	NA	NA	NA	4.38	300	1.38	2.39	0.29	0.29	0.03	9.7	0
2y2m	3.26	86	28	89	NA	NA	NA	NA	3.97	287	1.47	1.94	0.27	0.26	0.03	10.1	0.1
2y0m	3.74	83	31	102	NA	NA	NA	NA	6.3	271	2.7	2.9	0.3	0.3	0.1	NA	
reference range	4.0-5.4	70-86	32-41	105-140					5.0-15.0	150-500	1.0-7.0	2.0-8.0	0.3-1.3	<1.0	<0.3	8.0-12.0	0.00-0.6

Father Patient 1 (heterozygous)

Age	Erc count (x 10^6/µl)	MCV fl	Hkt %	Hb (g/L)	Reti (rel., %)	Reti (abs., x 10^3/µl)	Reti He (pg)	RPI	Neutrophils (x 10^3/µl)	Lymphocytes (x 10^3/µl)	Monocytes (x 10^3/µl)	Eosinophils (x 10^3/µl)	Basophils (x 10^3/µl)	Mean Platelet volume (fL)	Immature granulocytes (x 10^3/µl)	Mean Platelet volume (fL)2	Immature granulocytes (Tsd/µl)
44y11m	4.01	89.3	35.8	123	1.6	63.8	34.6	0.9	3.94	190	1.76	1.6	0.44	0.12	0.02	10.4	0.02
reference range	4.44-5.61	81.8-95.5	40-49.4	135-169	0.4-1.36	23-70.1	32.1-38.8	0.9	3.91-10.9	166-309	1.8-6.98	1.26-3.35	0.29-0.95	0.03-0.59	0.01-0.07	9.3-12.1	<0.06

Mother Patient 1 (heterozygous)

Age	Erc count (x 10^6/µl)	MCV fl	Hkt %	Hb (g/L)	Reti (rel., %)	Reti (abs., x 10^3/µl)	Reti He (pg)	RPI	Neutrophils (x 10^3/µl)	Lymphocytes (x 10^3/µl)	Monocytes (x 10^3/µl)	Eosinophils (x 10^3/µl)	Basophils (x 10^3/µl)	Mean Platelet volume (fL)	Immature granulocytes (x 10^3/µl)	Mean Platelet volume (fL)2	Immature granulocytes (Tsd/µl)
36y1m	4.12	92.2	38	124	1.8	75	35.3	1.2	6.73	274	3.9	2.06	0.56	0.19	0.02	10.4	0.03
reference range	3.92-5.08	82.9-98	36.6-44	119-146	0.4-1.36	17-63.8	32.1-38.8	2-3	4.49-12.68	173-390	2.1-8.89	1.26-3.35	0.25-0.84	0.01-0.4	0.01-0.07	9.1-11.9	< 0.06

Brother Patient 1 (heterozygous)

Age	Erc count (x 10^6/µl)	MCV fl	Hkt %	Hb (g/L)	Reti (rel., %)	Reti (abs., x 10^3/µl)	Reti He (pg)	RPI	Neutrophils (x 10^3/µl)	Lymphocytes (x 10^3/µl)	Monocytes (x 10^3/µl)	Eosinophils (x 10^3/µl)	Basophils (x 10^3/µl)	Mean Platelet volume (fL)	Immature granulocytes (x 10^3/µl)
13y6m	4.47	86.8	38.8	126	1.2	55.9	33.7	0.8	1.78	2.32	0.56	0.28	0.01	9.5	0.02
reference range	4.02-5.29	76.7-89.2	33.9-43.5	116-145	0.9-1.5	42-65	27-33.2	2-3	1.54-7.04	0.97-3.26	0.18-0.78	0.04-0.38	0.01-0.05	9.6-11.8	<0.03

Father Patient 3 (heterozygous)

Age	Erc count (x 10^{12}/L)	MCV (fL)	Hkt (%)	Hb (g/L)	Reti (rel., %)	Reti (abs., x 10^3/µL)	Reti He (pg)	RPI	Neutrophils (x 10^9/L)	Lymphocyctes (x 10^9/L)	Eosinophils (x 10^9/L)	Basophils (x 10^9/L)	Monocytes (x 10^9/L)
40y1m	4.8	85	41	141	N/A	N/A	N/A	N/A	2.37	1.64	0.08	0.02	0.35
reference range	4.3-5.75	80-99	39.5-50.5	135-172					1.5-7.7	1.1-4.5	0.02-0.5	0-0.2	0.1-0.9

Mother Patient 3 (heterozygous)

Age	Erc count (x 10^{12}/L)	MCV (fL)	Hkt (%)	Hb (g/L)	Reti (rel., %)	Reti (abs., x 10^3/µL)	Reti He (pg)	RPI	Neutrophils (x 10^9/L)	Lymphocyctes (x 10^9/L)	Eosinophils (x 10^9/L)	Basophils (x 10^9/L)	Monocytes (x 10^9/L)
33y11m	4.8	83	40	138	N/A	N/A	N/A	N/A	2.37	1.64	0.08	0.02	0.35
reference range	3.9-5.15	80-99	35.5-45	120-154					1.5-7.7	1.1-4.5	0.02-0.5	0-0.2	0.1-0.9

Sister A Patient 3 (heterozygous)

Age	Erc count (x 10^{12}/L)	MCV (fL)	Hkt (%)	Hb (g/L)	Reti (rel., %)	Reti (abs., x 10^3/µL)	Reti He (pg)	RPI	Neutrophils (x 10^9/L)	Lymphocyctes (x 10^9/L)	Eosinophils (x 10^9/L)	Basophils (x 10^9/L)	Monocytes (x 10^9/L)
15y1m	4.2	90	38	129	N/A	N/A	N/A	N/A	3.03	1.6	0.03	0.01	0.31
reference range	3.9-5.15	78-93	35.5-45	120-154					1.7-7.9	1.2-5.0	0.02-0.65	0-0.2	0.1-0.95

Sister B Patient 3 (heterozygous)

Age	Erc count (x 10^{12}/L)	MCV (fL)	Hkt (%)	Hb (g/L)	Reti (rel., %)	Reti (abs., x 10^3/µL)	Reti He (pg)	RPI	Neutrophils (x 10^9/L)	Lymphocyctes (x 10^9/L)	Eosinophils (x 10^9/L)	Basophils (x 10^9/L)	Monocytes (x 10^9/L)
13y11m	4.8	84	40	138	N/A	N/A	N/A	N/A	3.59	2.12	0.07	0.02	0.31
reference range	3.9-5.15	78-93	35.5-45	120-154					1.7-7.9	1.2-5.0	0.02-0.65	0-0.2	0.1-0.95

Sister C Patient 3 (heterozygous)

Age	Erc count (x 10^{12}/L)	MCV (fL)	Hkt (%)	Hb (g/L)	Reti (rel., %)	Reti (abs., x 10^3/µL)	Reti He (pg)	RPI	Neutrophils (x 10^9/L)	Lymphocyctes (x 10^9/L)	Eosinophils (x 10^9/L)	Basophils (x 10^9/L)	Monocytes (x 10^9/L)
12y2m	4.8	75	36	125	N/A	N/A	N/A	N/A	1.24	1.31	0.06	0.06	0.16
reference range	3.9-5.15	78-93	35.5-45	120-154					1.7-7.9	1.2-5.0	0.02-0.65	0-0.2	0.1-0.95

VI. Supplementary Table 5. Comparison of C112X *SOD1* homozygous iSODDES with D90A *SOD1* homozygous and G127X *SOD1* heterozygous ALS

	SOD1 genotype			
	C112X/C112X	C112X/wt	D90A /D90A	G127X/wt
Zygosity	hom	het	hom	het
Enzymatic activity in RBC	zero (devoid)	50%	normal	≈45%
Length of mutant protein	predicted truncated		normal length (?)	131 aa long
Hematological abnormalities	mild anemia with decreased glutathione	none	none	none
Gross liver & kidney blood parameters	normal	normal	normal	normal
Skin fibroblasts in culture	only grow when PO2 is reduced to 2% and with ascorbate in the culture medium	normal growth	easily grown	normal growth
Mutant SOD1 protein detectable in fibroblasts	only with proteasome inhibition	only with proteasome inhibition	Yes	Yes
Mutant SOD1 aggregation detectable in fibroblasts	no	no	no	only with proteasome inhibition
Age at onset of motor symptoms	6-12 months of age	none	20-94 years median 47 years	≈50 years
First symptom(s) observed	motor	none	frequently neuralgic lumbago, then motor	motor only
First reported motor symptom or sign	hypotonia of truncal muscles	none	asymmetrical increased muscle tone and brisk reflexes in one leg, later also in the opposite leg before ascending to the upper limbs	asymmetrical LMN & UMN paresis in a limb or truncal innervated muscle
Second motor symptom/sign	increased muscle tone and brisk DTR's in the limbs, probably first in the legs (?)	none	LMN involvement then dominates first in the legs, later also in the arms and bulbar innervated muscles	spread from one limb to all limbs and bulbar innervated muscles
Bulbar involvement	yes and early, UMN dominant	none	yes but late, both UMN and LMN with severe tongue atrophy	yes, early LMN dominant
Affective lability (in all mild)	yes, first noted >10 y after onset	none	yes, >10 years after onset of motor symptoms	yes
Gross motor disease progression estimate	fast, then slow?	n a	invariably very slow	fast
Survival time from onset	?	(normal life span?)	14.2 years	2.8 years
Neurological phenotype	uniform	(uniform in the sense that none of them have an MND or other distinct neurological phenotype, n = 10 with the oldest being <45 yeras old)	strictly uniform for site of onset and disease progtression rate	highly variable for site of onset but invariably rapid progression
Urinary bladder involvement	urgency of micturition 4/8 patients	no	frequently urgency of micturition (also observed in tg-D90A hSOD1 mice model)	no
Non-neurological findings	yes	no	no	no
Cortical atrophy of the cerebrum	yes	not studied	no	no
Progressive vermis cerebelly atrophy	yes	not studied	no	no
Brain stem atrophy on MRI	yes	not studied	no	no
Source:	this report	this report	Modified from Andersen PM et al., Brain 1996;119:1153-1172	Modified from Andersen PM et al., Brain 1997;120:1723-1737

DTR, deep tendon stretch reflexes

LMN, lower motor neuron ("secondary" motor neuron)

UMN, upper motor neuron ("first" motror neuron) and associated tracts

aa, amino acids

VII. Supplementary Methods

Immunoblots of fibroblast cultures heterozygous for SOD1 p.C112Wfs*11

Fibroblast cell lines from the mother (M2) and father (F2) of patient 2 were established. We used in house generated fibroblast lines derived from WT controls and ALS patients carrying the ALS-associated truncation mutation p.G127X in SOD1 for comparison. Fibroblasts were cultured to 70-80% confluency, and incubated for 24 hours with, or without, bortezomib 5 ng/ml before harvest and storage of cell pellets at -80°C. Quantification of soluble misfolded SOD1 was done following a published protocol [3] on cells harvested with 40 mM iodacetamide to prevent any oxidation of reduced cysteines to disulfide bridges and refolding during sample preparation. Cell lysates or hemolysates were loaded on Any kD Stain free Criterion TGX precast gels. Gels were activated and protein load images captured using a Geldoc imager, before blotting on nitrocellulose filters. Primary rabbit anti-human SOD1 antibodies raised against peptides corresponding to aa 25-40 (1 µg/ml) or aa 5-21 (1 µg/ml) and were analyzed by western blotting using Any kD Criterion TGX precast gels (BioRad Laboratories, Hercules, CA, USA). To allow specific detection of the C112X mutant protein, we used a rabbit polyclonal antibody against a peptide corresponding to the first 8 aa:s (italics*)* of the 11-aa long neo-peptide ***GDHWHHWP***HTG in the C-terminal end of the C112X neo-peptide (10 µg/ml) [2]. The G127X protein was detected using an antibody generated in the same way using a peptide corresponding to the C-terminal end, including aa 123–127 of the SOD1 sequence and the GQRWK neo-peptide (2ug/ml) [5,6]. Horseradish peroxidase (HRP)-conjugated secondary anti-rabbit IgG; 1:10,000, Dako, Glostrup, Denmark). ECL Select reagent (GE Healthcare Biosciences, Chicago, IL, USA) was used to detect the signal. Images were acquired using a Chemidoc apparatus and analyzed using ImageLab software.

LC-MS analysis of GSH, GSSG and ascorbate

Following initial sample preparation as outlined in the main manuscript, all mixed samples were then homogenized in a bead mill (Retsch MM400) at 30 oscillations/s for 1 min, followed by centrifugation at 14 000 rpm for 20 minutes (Hettich Zentrifugen). 150 µL of the supernatant was transferred to a spin filter (Ultrafree MC, 0.22 µm, Millipore, Burlington, MA, USA) and centrifuged at + 4˚C, 14000 rpm for 10 min to remove any remaining protein precipitate. 35 µL of the filtered extract was transferred to an LC-MS vial and further diluted with 65 µL of 2.5% MPA prior to LC-MS analysis (the final concentration of MPA in the vial was 2.5%). 1µL was injected and analyzed by LC-ESI-MSMS on a 1290 Infinity system from Agilent Technologies (Waldbronn, Germany) coupled to an Agilent 6490 Triple quadrupole mass spectrometer. The chromatographic separation was achieved using an Acquity UPLC HSS T3 column (2.1 x 100 mm, particle size 1.8 µm), thermostated at 40 °C. Analyses were performed as previously described (4, 5).

VIII. Uncropped blots for Figures 3 and 4

A. Uncropped blots and gel for Fig. 3

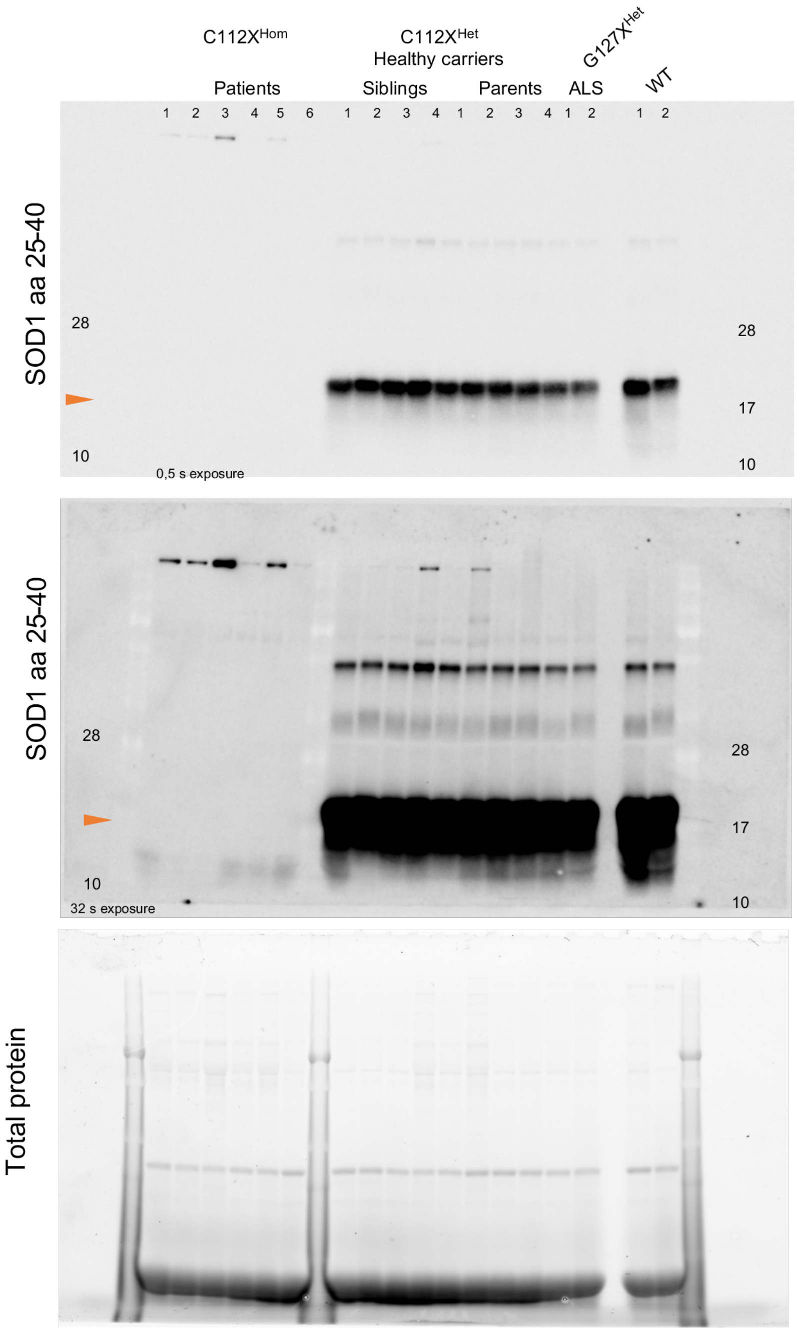

Red arrow indicates mobility of SOD1 C112X truncation variant.

B. Uncropped blots for Figure 4

Soluble fraction

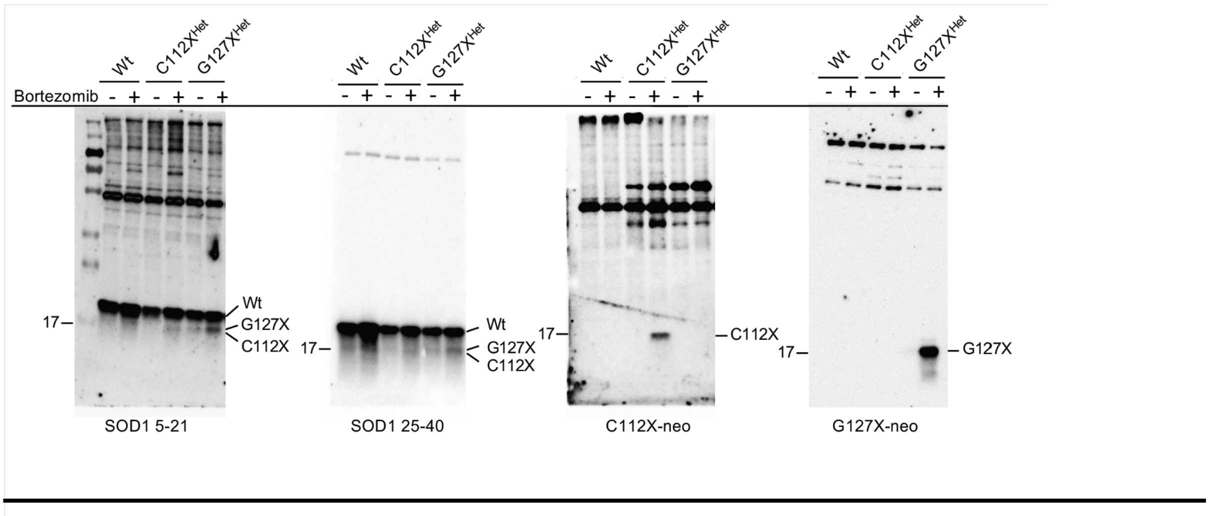

Insoluble fraction

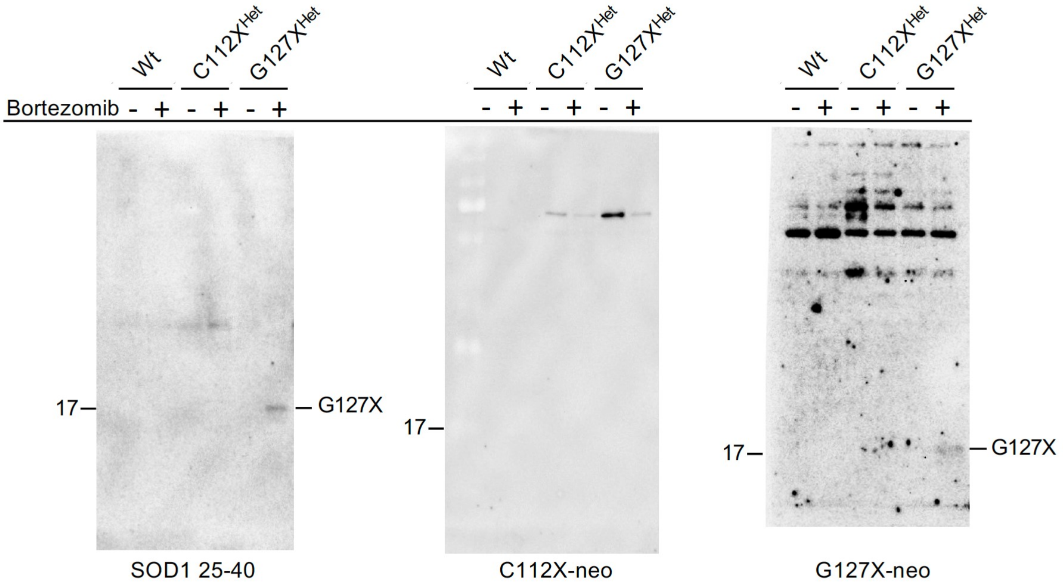

IX. References

1. Park JH, Elpers C, Reunert J, et al. SOD1 deficiency: a novel syndrome distinct from amyotrophic lateral sclerosis. *Brain*. 2019;142(8):2230-2237.

2. Andersen PM, Nordstrom U, Tsiakas K, et al. Phenotype in an Infant with SOD1 Homozygous Truncating Mutation. *N Engl J Med*. 2019;381(5):486-488.

3. Keskin I, Forsgren E, Lehmann M, et al. The molecular pathogenesis of superoxide dismutase 1-linked ALS is promoted by low oxygen tension. *Acta Neuropathol*. 2019;138(1):85-101.

4. Ekhtiari Bidhendi E, Bergh J, Zetterstrom P, et al. Mutant superoxide dismutase aggregates from human spinal cord transmit amyotrophic lateral sclerosis. *Acta Neuropathol*. 2018;136(6):939953.

5. Claeson AS, Gouveia-Figueira S, Stenlund H, Johansson AI. A standardized protocol for comparable analysis of GSH/GSSG by UHPLC-ESI-MSMS for human plasma. *J Chromatogr B Analyt Technol Biomed Life Sci*. 2019;1104:67-72.

6. Cao L, Waldon D, Teffera Y, et al. Ratios of biliary glutathione disulfide (GSSG) to glutathione (GSH): a potential index to screen drug-induced hepatic oxidative stress in rats and mice. *Anal Bioanal Chem*. 2013;405(8):2635-2642.
